# Social Determinants of the Diabetes–Depression Dyad Within a Syndemic Framework: A Scoping Review

**DOI:** 10.3390/nursrep16100360

**Published:** 2026-10-12

**Authors:** Diana Patricia Londoño Buriticá, Brenda Yuliana Herrera-Serna, Consuelo Vélez Álvarez

**Affiliations:** 1 Faculty of Health Sciences, Universidad del Quindío, Carrera 15 Calle 12 Norte, Armenia 630004, Quindío, Colombia; dplondono@uniquindio.edu.co; 2 Doctoral Program in Health Sciences, Universidad Autónoma de Manizales, Manizales 170001, Caldas, Colombia; 3 Oral Health Department, Faculty of Health, Universidad Autónoma de Manizales, Manizales 170001, Caldas, Colombia; 4 Public Health Department, Faculty of Health, Universidad Autónoma de Manizales, Manizales 170001, Caldas, Colombia; cva@autonoma.edu.co

**Keywords:** syndemic, diabetes, depression, social determinants of health, nursing

## Abstract

**Introduction:** A syndemic is characterised by the co-occurrence, clustering, and adverse interaction of multiple diseases in populations affected by social disadvantage. The diabetes–depression dyad is increasingly examined through this lens, yet its social determinants remain fragmented and rarely translated into nursing-relevant guidance for marginalised populations. This scoping review aimed to map the structural and intermediary social factors associated with the diabetes–depression dyad within the syndemic framework and to describe how biosocial interaction has been conceptualised and operationalised. **Methods:** Following the Joanna Briggs Institute methodology and reporting using PRISMA-ScR, we searched Scopus, Web of Science, PubMed, ScienceDirect, CINAHL, PsycINFO, Embase and Google Scholar up to 20 September 2026, with no restrictions on language, design, or year. We interpreted findings using an adapted VIDDA framework aligned with the WHO model of Social Determinants of Health. **Results:** Thirty-five publications (2012–2026) were included. Structural violence, poverty, displacement and migration were the most frequently reported structural determinants, whereas access to health services, neighbourhood conditions and interpersonal abuse were the most frequent intermediary factors. Biosocial interaction was formally tested in only a minority of studies, mainly through additive or multiplicative models; most publications documented co-occurrence or association rather than demonstrated synergy. **Conclusions:** Social determinants are central to the diabetes–depression syndemic in vulnerable groups, yet the evidence remains conceptually rich but empirically uneven, with few studies formally testing biosocial interaction. For nursing practice, these findings support pairing routine glycaemic and mood assessment with structured screening for social needs, coordinating integrated care, and delivering culturally safe, person-centred support. The included studies did not directly evaluate whether such nurse-led, socially informed care reduces health inequities; longitudinal and interventional research is needed to test this.

## 1. Introduction

Diabetes and depression frequently co-occur. Meta-analytic evidence indicates roughly twofold higher odds of prevalent depression among people with diabetes, while longitudinal studies estimate a 23–27% higher risk of developing depression [1]. Depression is associated with poorer diabetes self-care and lower adherence to diet and physical-activity recommendations, although intervention evidence remains inconsistent [2]. In low- and middle-income countries, a recent meta-analysis also linked depression with poorer glycaemic control; substantial between-study heterogeneity means the magnitude of that association varies by setting and diagnostic approach [3]. Longitudinal evidence links depression with subsequently higher rates of macrovascular and microvascular complications, though cohort bias cannot be fully excluded [4]. A systematic review comparing diabetes types found higher depression prevalence in both type 1 (22% vs. 13%) and type 2 diabetes (19% vs. 11%) than in people without diabetes [5]. Estimates were highest in specialist-care samples and showed a stronger relative association in low- and middle-income settings, supporting systematic assessment of depression and diabetes distress as part of chronic-disease care.

Beyond cross-sectional associations, longitudinal evidence supports reciprocal associations between diabetes, depression, and diabetes outcomes. A meta-analysis of longitudinal cohorts found that baseline depression was associated with later macrovascular and microvascular complications; existing complications also predicted subsequent depressive disorder, although the underlying observational studies had some risk of bias [4]. A separate systematic review found small bidirectional associations between depressive symptoms and HbA1c over roughly two years of follow-up: higher baseline depressive symptoms predicted slightly higher subsequent HbA1c, and higher baseline HbA1c predicted an 18% higher likelihood of probable depression. The authors therefore proposed concurrent attention to mood and glycaemic control, while noting that larger longitudinal studies are needed to confirm the association [6].

From an epidemiological standpoint, Global Burden of Disease (GBD) analyses identify diabetes and depressive disorders as major contributors to global health loss. In 2023, GBD data estimated 529 million people living with diabetes worldwide, with diabetes prevalence increasing globally since 2000; type 2 diabetes accounted for most diabetes-related disability-adjusted life years [7]. Depressive disorders likewise imposed a substantial absolute burden, with an estimated 332 million prevalent cases and 56 million DALYs in 2021 [8]. The comorbidity is plausibly concentrated where social disadvantage is greatest. Still, the evidence should be stated cautiously: a review found few studies that reported evidence that depression among people with diabetes was associated with lower socioeconomic status [9]. Low household income, adverse childhood experiences, ethnic discrimination, and neighbourhood problems were related to comorbid diabetes and depression in U.S. Latinos, using a syndemic framework [10].

Drawing on anthropological work on HIV/AIDS, substance use, and health inequality, Merrill Singer and colleagues developed the concept of syndemics to describe the synergistic interaction of co-occurring diseases and the social conditions that intensify their combined burden [11]. Syndemic theory focuses on three linked questions: why diseases cluster in particular populations; how they interact biologically and behaviourally; and how social environments, including inequality and injustice, shape vulnerability, clustering, and interaction. This lens has been applied to diabetes and depression, particularly in Emily Mendenhall’s VIDDA framework—Violence, Immigration, Depression, Diabetes, and Abuse. Developed from life-history, survey, psychiatric, and biological data from low-income Mexican immigrant women in Chicago, VIDDA describes how structural and interpersonal violence, migration-related isolation, poverty, and abuse can intersect with diabetes and depression in that specific setting [12,13].

Multimorbidity research and diabetes–depression studies support care models that address mental and physical health together, particularly where social disadvantage increases barriers to self-management and access to care. Evidence from low- and middle-income settings also points to the need for mental-health care that is integrated into diabetes services. However, prevalence estimates vary with sampling and measurement methods [14]. At the clinical level, collaborative and integrated care reliably improves depressive outcomes and may improve adherence. Effects on HbA1c are less uniform: earlier collaborative-care meta-analyses found no statistically significant glycaemic advantage, whereas a broader 2024 synthesis of 31 trials reported modest improvements in both HbA1c and depressive symptoms, with substantial heterogeneity across intervention types [15]. Technology-supported models are increasingly being used to connect diabetes and mental-health care, but the evidence base remains heterogeneous, and gaps persist in professional integration and implementation capability [16]. These findings favour integrated, patient-centred care over siloed delivery, while leaving the optimal configuration and the contribution of components such as co-location, stepped care, or digital support unresolved.

Because the diabetes–depression dyad clusters in populations affected by poverty, violence, migration and constrained access to care, it is best understood as a problem of health equity rather than of clinical management alone [10,11]. Marginalised groups experience the syndemic not only as two interacting diseases but as an accumulation of social disadvantage that shapes exposure, self-management and the ability to reach and remain in care [12,13]. Addressing such conditions is central to contemporary nursing science, which increasingly frames chronic-illness care through the social determinants of health and person-centred culturally safe practice [17].

Nurses provide continuous, longitudinal contact across primary, community and hospital settings. They are therefore strategically placed to detect the social conditions that drive the diabetes–depression syndemic, to coordinate care across fragmented services, and to deliver interventions that address both mental and physical health together [14,16]. However, the evidence linking chronic-disease trajectories to social determinants has largely developed within medical and epidemiological traditions, and nursing-led or nursing-focused contributions remain scarce [18]. Mapping how structural and intermediary social factors have been studied in this dyad is thus a necessary step to inform equity-oriented nursing practice for vulnerable populations.

In this context, syndemic thinking provides a useful lens for linking the global burden of diabetes and depression, their reciprocal clinical associations, and the social conditions under which multimorbidity and care fragmentation occur [8,19,20]. By locating the diabetes–depression dyad within structural and intermediary determinants of health, this perspective directs attention beyond biological mechanisms and clinical management to living conditions, social position, and access to coordinated medical and psychosocial services. Evidence supports this as a plausible organising framework, but causal pathways remain incompletely tested across settings [10].

Several authors argue that healthcare systems should move beyond isolated disease management toward coordinated medical, psychosocial, and intersectoral strategies that address the social conditions associated with disease clustering and fragmented care [16,19]. From this perspective, syndemic thinking reframes diabetes–depression care by recognising that clinical outcomes and treatment experiences may reflect not only biological and behavioural interactions between the two conditions, but also structural and intermediary determinants—including social position, living conditions, psychosocial stress, and access to coordinated services [10]. Informed by the original VIDDA framework [12,13] and aligned with the WHO model of Social Determinants of Health [21], the present review applies and advances an adapted framework; the VIDDA–WHO SDH adaptation (Figure 1) is presented as an explicit analytical contribution rather than a purely illustrative diagram. This adaptation reorganises VIDDA’s original elements (violence, immigration-related stress, depression, diabetes, and abuse) into two operational domains of the WHO framework—structural and intermediary determinants—linked to the syndemic core (the diabetes–depression dyad and its interacting comorbidities) through biosocial interaction and provides the a priori structure used to chart, classify, and interpret the evidence throughout this review.

Given that research on the diabetes–depression dyad through a syndemic lens is conceptually evolving and spans qualitative, quantitative, and mixed-methods traditions, a scoping review is an appropriate design. Scoping reviews systematically identify and map the breadth of evidence on a topic, clarify key concepts and definitions, and describe research characteristics across diverse sources and contexts [22]. This approach is particularly suited to examining how structural and intermediary social factors have been studied in relation to the diabetes–depression dyad and how biosocial interaction has been conceptualised and operationalised. It can also identify knowledge gaps and inform whether more focused systematic reviews are warranted [23,24]. The review therefore maps and characterises the available literature rather than producing a pooled estimate of intervention effectiveness or association strength.

Beyond its epidemiological burden, the diabetes–depression dyad is increasingly managed at the level of primary and community care, where nurses are often the first and most continuous point of contact. Nurse-led models have shown tangible clinical value in type 2 diabetes: community-based, nurse-led support interventions can reduce HbA1c [25], and lifestyle-medicine case-manager nursing roles have been proposed as a structured framework for long-term metabolic and behavioural management [26]. At the same time, outcomes depend on factors that are explicitly social and communicative—diabetes distress, health literacy, the quality of diabetes education and patient–provider communication are all associated with self-care [27]—which situates the diabetes–depression dyad within, rather than apart from, the social determinants of health.

Prior reviews have largely examined diabetes and depression either as separate conditions or as a clinical comorbidity, with less attention to how structural and intermediary social determinants shape their co-occurrence through a syndemic lens, and with limited translation into nursing practice. This review addresses that gap. Its original contribution is to map, within an explicitly syndemic framework, the social factors associated with the diabetes–depression dyad and how biosocial interaction has been conceptualised and tested as well as to connect that map to concrete, nurse-relevant points of action across screening, integrated care and intersectoral coordination.

The primary objective of this scoping review is to map the characteristics, distribution, and thematic focus of research examining the diabetes–depression dyad through a syndemic lens, with particular attention to structural and intermediary social factors. Accordingly, this review maps evidence framed within the syndemic perspective rather than the entire literature on diabetes, depression, and social disadvantage; this eligibility choice provides conceptual coherence and is revisited as a limitation. The secondary objective is to describe how this literature has conceptualised and operationalised biosocial interaction. Consistent with the Joanna Briggs Institute Population–Concept–Context (PCC) framework, the review asks the following: (1) Which structural and intermediary social factors have been investigated in relation to the diabetes–depression dyad and (2) how have studies conceptualised and empirically assessed interaction within syndemic research on the diabetes–depression dyad?

## 2. Methods

This scoping review mapped available evidence on social factors associated with the diabetes–depression dyad within the syndemic theoretical framework. It examined how the literature has conceptualised and operationalised the principle of biosocial interaction. The review design was selected because the field is methodologically heterogeneous, conceptually evolving, and includes qualitative, quantitative, mixed-methods, review, and grey-literature sources, making a scoping approach more appropriate than an effect-size–oriented systematic review. The review followed the methodological framework proposed by Arksey and O’Malley and the updated Joanna Briggs Institute (JBI) guidance for scoping reviews [24,28].

The review questions followed the JBI-recommended PCC framework. The population included individuals with diabetes mellitus and/or depression, without restrictions on age, sex, or geographic setting. The concept of interest was the syndemic clustering of the diabetes–depression dyad, including associated structural and intermediary social factors and the models used to examine biosocial interaction. The context comprised publications from any country, with no restrictions on study design, language, or year of publication, if they explicitly referred to the syndemic theoretical framework and were available up to 20 September 2026.

### 2.1. Protocol Registration and Methodological Transparency

As a transparency measure, we deposited a review protocol on the Open Science Framework (OSF) on 16 April 2026 (https://doi.org/10.17605/OSF.IO/24GNB); the record was last updated on 3 May 2026. The protocol was deposited after the review had begun and is therefore not a prospective registration. At the time of deposit, the initial database searches (February 2026) had been run, and title/abstract screening had started; full-text eligibility assessment and data charting had not been completed. All searches were re-run on 20 September 2026. The review questions, eligibility criteria and scope were not modified after deposit. Changes to the set of included sources that resulted from the search update are reported in Section 3. Because the searches were updated on 20 September 2026—after the protocol was deposited (16 April 2026) and last amended (3 May 2026)—we treat this update as a currency (date-limited) search rather than a change of methods: the review questions, eligibility criteria, data-charting form and analysis plan were unchanged, and only the search date and the resulting set of records were affected. We made the OSF record publicly accessible and amended it to document the 20 September 2026 search update, so the deposited protocol and the methods reported here are consistent.

### 2.2. Information Sources and Search Strategy

We developed the search strategy in collaboration with a health sciences librarian to maximise sensitivity for publications explicitly addressing syndemics in relation to diabetes and depression. We searched the following electronic sources: PubMed, Scopus, Web of Science, ScienceDirect, CINAHL, PsycINFO, and Embase. To broaden retrieval beyond the indexed literature, we also searched Google Scholar for grey literature and potentially relevant non-indexed documents. We restricted the Google Scholar advanced search to the article title (terms “syndemic” AND “diabetes” AND “depression”; citations excluded), which returned 9 records, all of which we screened; no further stopping rule was required. We searched ProQuest Dissertations & Theses, accessed through an institutional subscription, with the same terms; the search returned 1 record, which we screened. We first ran the searches in February 2026 and re-ran all of them in full on 20 September 2026; the numbers reported here are those of the second run. We applied no restrictions on language, publication date, or study design.

The strategy combined controlled vocabulary and free-text terms related to three core concepts: syndemics, diabetes, and depression. In PubMed, we used MeSH terms and title/abstract terms. In the other databases, we adapted the syntax to each platform’s search structure and controlled vocabulary where applicable. The Supplementary Material presents the complete search strategies for all databases, including platform-specific syntax, search date, filters applied, and number of records retrieved. An example of the PubMed strategy is provided below:

(“Syndemics”[MeSH Terms] OR “Syndemic”[tiab] OR “Syndemic theory”[tiab] OR “Syndemic framework”[tiab] OR “Syndemics”[tiab])
AND
(“Diabetes Mellitus”[MeSH Terms] OR “Diabetes Mellitus, Type 2”[MeSH Terms] OR “Diabetes”[tiab] OR “Type 2 diabetes”[tiab] OR “T2DM”[tiab])
AND
(“Depression”[MeSH Terms] OR “Depressive Disorder”[MeSH Terms] OR “Depressive Disorder, Major”[MeSH Terms] OR “Depression”[tiab] OR “Depressive symptoms”[tiab] OR “Depressive disorder”[tiab] OR “Mental health”[tiab])

The searches retrieved 219 records: 209 from databases (26 PubMed, 67 Web of Science, 41 Scopus, 55 ScienceDirect, 3 CINAHL, 0 PsycINFO, and 17 Embase) and 10 from registers (9 Google Scholar and 1 thesis), as detailed in the Supplementary Material.

### 2.3. Eligibility Criteria

Publications were eligible for inclusion if they met all the following criteria:

1. Full-text availability;
2. Explicit reference to the coexistence of diabetes and depression;
3. Explicit use of the syndemic theoretical framework (e.g., “syndemic”, “VIDDA”, biosocial interaction) as a conceptual or analytical lens;
4. Empirical or conceptual contribution to the understanding of social factors or biosocial interaction in the diabetes–depression dyad.

We assessed criteria 2 and 3 on the full text of each publication, not on its title, abstract or indexing terms. In keeping with the mapping purpose of a scoping review, we applied both criteria inclusively and recorded the level at which each publication met them. Criterion 2 was met directly when the publication explicitly addressed diabetes and depression as coexisting or interacting conditions, whether as its main focus or as one of the disease clusters it analysed or reviewed. It was met indirectly when the publication addressed both conditions but their coexistence was not the subject of explicit analysis: for example, when one of them was a component of a wider multimorbidity cluster or analytical model, was an adjustment variable, or was discussed as part of the syndemic context without being analysed, or when the two conditions were examined in separate case studies. Criterion 3 was met directly when syndemic theory was the stated conceptual or analytical framework and met indirectly when syndemic concepts were used to frame or interpret the findings without structuring the analysis. Publications that met a criterion only indirectly were retained because they show how the dyad appears within broader syndemic research. They are identified in Table 1 and Supplementary Table S1 and are treated as contextual, not core, evidence.

Empirical studies included quantitative, qualitative, and mixed-methods designs (e.g., cohort studies, cross-sectional analyses, ethnographic work, case studies, surveys). Conceptual and narrative contributions included review articles, theoretical papers, books, and theses that used syndemic theory to discuss diabetes and depression even when they did not present primary data. We included grey literature (e.g., reports, theses) when it met the criteria above and provided sufficient methodological detail.

The deliberate inclusion of grey and non-peer-reviewed sources (books, theses, editorials, and short commentaries) reflects the mapping purpose of a scoping review, which seeks to capture the full breadth and conceptual development of a field rather than to appraise or pool effect estimates [22,28]. Because syndemic theory has advanced substantially through book-length ethnographies and doctoral work—most notably the foundational contributions that developed the VIDDA framework—restricting eligibility to indexed, peer-reviewed articles would have excluded primary sources central to how the diabetes–depression syndemic has been conceptualised. This choice contrasts with reviews that limit inclusion to peer-reviewed studies to reduce methodological heterogeneity; here, we prioritised comprehensive conceptual coverage, consistent with JBI guidance for scoping reviews [22]. To mitigate these risks, we recorded the source type for each item. We applied analytic safeguards (Section 2.6 and Section 3), so conceptual and non-peer-reviewed sources informed interpretation without inflating quantitative counts.

We applied no restrictions on language, publication year, or geographic setting. Publications were excluded if they: (a) consisted only of conference abstracts or oral presentations without accessible full text; (b) addressed diabetes or depression separately without a syndemic perspective; or (c) discussed multimorbidity or comorbidity in general without explicitly engaging the syndemic concept.

### 2.4. Operational Definition of Structural and Intermediary Social Factors

The VIDDA–WHO SDH adaptation was operationalised a priori in two domains, which structured the entire charting process. Structural determinants captured VIDDA’s attention to violence and migration within the WHO categories of socioeconomic and political context, socioeconomic position, and structural/symbolic discrimination. Intermediary determinants captured how these forces manifest in everyday life through material circumstances, psychosocial stressors, behavioural and biological mediators, and health-system factors. The syndemic core elements (diabetes, depression, and interacting comorbidities) and their biosocial interaction constituted the outcome around which both domains were mapped.

Structural factors were understood as macro-level and positional determinants and included the following:

- Policy and governance (macroeconomic policies, social protection, health policy, political violence, migration regimes);
- Socioeconomic position (education, occupation, income, wealth, social class, gender, race/ethnicity);
- Structural and symbolic discrimination (structural violence, racism, sexism, homophobia, intersectional stigma).

Intermediary factors were defined as the conditions through which structural determinants are expressed in everyday life and include the following:

- Material circumstances (housing quality, food insecurity, neighbourhood safety and environmental conditions);
- Psychosocial stressors (interpersonal abuse, family conflict, social isolation, coping resources, illness-related distress);
- care access and health system factors (availability, affordability and continuity of medical and mental-health care, barriers to treatment adherence);
- Behavioural and biological mediators (health-related behaviours, substance use, functional disability), when explicitly linked to social context.

We applied these definitions consistently when charting data. When a factor could plausibly fit both domains (e.g., neighbourhood characteristics), we followed the WHO SDH framework and the authors’ conceptualisation in the original publication as the primary classification. We documented any ambiguity in the data charting form.

### 2.5. Study Selection

We compiled all retrieved references and screened for duplicates. After removing duplicates, two reviewers (D.P.L.B. and B.Y.H.-S.) independently screened titles and abstracts. The same reviewers then independently assessed full-text eligibility. The reviewers resolved disagreements by discussion and, when needed, by consulting a third reviewer (C.V.Á.). The PRISMA flow diagram in the Results section presents the study identification and selection process.

To verify that the included evidence conformed to the eligibility criteria, two reviewers independently audited the full text of all 35 included publications against criteria 2 and 3. Supplementary Table S1 reports, for each publication, the level at which each criterion was met (direct or indirect), the supporting passage and its page. Twenty-eight publications met both criteria directly and seven met one of them indirectly.

### 2.6. Data Charting and Synthesis

We developed a standardised data charting form a priori and piloted it on five studies before full extraction. For each included publication, we recorded first author, year of publication, country, study design, sample size, study population, comorbidities explored, type of contribution (empirical quantitative, empirical qualitative/mixed, conceptual/narrative), structural social factors, intermediary social factors, and statistical or conceptual approaches used to assess biosocial interaction.

Data charting and VIDDA–WHO classification were performed independently by two reviewers (D.P.L.B. and B.Y.H.-S.) and cross-checked; disagreements were resolved by discussion with a third reviewer (C.V.Á.), who is a recognised author on the social determinants of health. After the five-study pilot, we refined the charting form wording only, with no changes to the extracted domains.

We extracted and coded social factors according to the operational definitions of the structural and intermediary determinants described above, based on the WHO SDH framework and the VIDDA adaptation. When a publication used its own terminology (e.g., “syndemic suffering”, “social vulnerability”), we mapped the underlying elements onto the structural/intermediary categories. We noted publications that formally examined interaction through additive or multiplicative statistical models, which we later summarised separately.

Given the objectives of a scoping review, the synthesis was descriptive and mapping-oriented rather than effect-size based. We organised findings by publication type (empirical vs. conceptual), comorbidities examined, structural and intermediary factors identified, and approaches used to conceptualise or test biosocial interaction (e.g., quantitative interaction models, qualitative narratives, theoretical elaborations).

### 2.7. Quality Appraisal

In accordance with JBI guidance for scoping reviews, we did not conduct a formal methodological quality appraisal of the included studies. This decision aligns with the purpose of scoping reviews, which map the breadth and nature of available evidence rather than exclude studies based on methodological quality. Nevertheless, we report key methodological characteristics of the included studies to help readers assess the heterogeneity and evidentiary profile of the literature reviewed.

## 3. Results

The updated search (20 September 2026) identified 219 records. After removing 81 duplicates, 138 records were screened at title and abstract, of which 90 were excluded as not relevant to the review question. The remaining 48 reports were sought and assessed in full text (none were unretrievable); 13 were excluded—12 because they did not address the coexistence of or interaction between diabetes and depression, and 1 because it was not relevant to the syndemic concept—leaving 35 publications included, in exact correspondence with the PRISMA-ScR flow diagram (Figure 2). Compared with the initial February 2026 search, the September 2026 currency update did not add new sources to the evidence base; for some theses identified in the initial search, the corresponding peer-reviewed articles had since been published and were therefore counted as original articles rather than theses. This reclassification affected only the document-type counts, not the number or identity of the included studies, which remained 35. Of the 35 publications ultimately included, 34 (97.1%) were written in English and one (2.9%) in Italian with an English abstract [29] all were published from 2012 onwards. WOS had the most publications (*n* = 67). Figure 2 shows the identification and selection scheme following the PRISMA 2020 framework for scoping reviews [24]. Table 1 presents the publications included in the review, arranged by document type and, subsequently, alphabetically by the first author’s last name and year of publication (starting with the most recent), and summarises their main characteristics.

This review shows that scientific production is concentrated in a few countries, with the United States standing out; in South America, Brazil has produced two scientific articles. More broadly, several publications share an underlying study or dataset (Table 1 and Table 2: Brazil National Health Survey [31,32]; Chicago/VIDDA data [12,13,33]; and the Johannesburg Bt20 cohort [13,34,35,36]); these were retained as separate publications but treated cautiously so as not to be read as independent evidence.

Seven of the 35 publications included met one core eligibility criterion only indirectly (Supplementary Table S1). Without them, every category of social determinant in Table 2 remains populated, with no row losing more than four publications. Four of the five studies that formally tested biosocial interaction (Table 3) are unaffected; the fifth [37] tested the interaction between diabetes and hypertension, not between diabetes and depression.

Between the original submission and the updated search, the set of included publications changed in three respects: one doctoral thesis was removed because the article reporting the same study was already included [38]; a second thesis was replaced by the peer-reviewed article derived from it [39]; and one qualitative article identified in the updated search was added [40].

Although scientific contributions on the diabetes–depression syndemic have been documented since 2012, publications have increased over the past 6 years (57.14% of the total output). Initially, exploratory studies used a qualitative approach based on ethnographic data [12,13,33,34,35,36,40,41,42]. Since 2014, mixed-methods studies have been developed [13,34,35,36,43,44], and by 2019, population-based research had become established, using databases from countries and even regions worldwide [31,32,37,39,45]. Since 2017, narrative and scoping reviews have been identified, with the highest output in 2023 [9,46,47,48,49]. Table 1 shows the characteristics of the included studies.

**Table 1:** Characteristics of the included studies.

Study [Ref]	Year	Country/Setting	Population and Sample (*n*)	Comorbidities Examined	Design/Data Source	Evidence Type
**Review Studies (*n* = 5)**						
Gizamba et al. [46]	2023	—	29 documents	Multimorbidity (review of syndemics research)	Systematic review (MEDLINE, Embase); records 2017–2022	Review (secondary)
Mendenhall et al. [49]	2017	India, Kenya, South Africa	—	Diabetes, HIV, Depression, TB	Review	Review (secondary)
Perez et al. [9]	2024	—	—	Diabetes, Depression, Inflammatory markers	Scoping review (PubMed, CINAHL, PsycINFO, WOS); 2006–2022	Review (secondary)
Saqib et al. [47]	2023	—	71 documents	COVID-19, Anxiety, Depression, Diabetes, Hypertension, Asthma, CHD	Narrative review (PsycINFO, PubMed, Google Scholar, WOS)	Review (secondary)
Singer et al. [48]	2020	—	143 documents	Multimorbidity (review of syndemics research)	Systematic review (AnthroSource, CINAHL, Google Scholar, PubMed, Scopus)	Review (secondary)
**Original Articles (*n* = 21)**						
Agudelo-Botero et al. [45]	2022	Mexico	Adults > 20 y; *n* = 16,835	Type 2 diabetes, Depression, Obesity	Observational (ENSANUT) 2018–2019	Empirical—quantitative
Byg et al. [50]	2016	USA (Boston)	MSM ≥ 18 y with diabetes and HIV; *n* = 88	Diabetes, HIV, Hypertension, Depression, Substance abuse	Retrospective (medical records)	Empirical—quantitative
Chichetto et al. [51] †	2019	USA	Veterans with HIV, matched uninfected; *n* = 6721	Depressive symptoms, Unhealthy alcohol use, Cigarette use, HIV; All-cause mortality; Diabetes (adjustment variable)	Cohort (VACS)	Empirical—quantitative
Dansero et al. [39]	2026	Italy (Piedmont)	Men and women 45–75 y; *n* = 1,778,845	Diabetes, Depression	Population-based cohort (Piedmont Longitudinal Study); LCA + Cox	Empirical—quantitative
Diderichsen et al. [31] ^A^	2021	Brazil	Adults ≥ 18 y; 81,357 households/60,202 individuals	Disability, Diabetes, Depression	National Health Survey 2013	Empirical—quantitative
Diderichsen and Andersen [32] ^A^	2019	Brazil	Adults ≥ 18 y; 81,357 households/60,202 individuals	Diabetes, Depression	National Health Survey 2013	Empirical—quantitative
Eshak et al. [52]	2024	USA (Pennsylvania)	Adults ≥ 18 y with HIV; *n* = 621	Depression, Diabetes, HIV, HIV treatment retention	Retrospective (medical records)	Empirical—quantitative
Gupta et al. [53] †	2022	Canada (New Brunswick)	Diabetic adults ≥ 19 y; *n* = 66,275	Diabetes, Depression, Mood disorders, Anxiety	Observational cohort (6 y follow-up)	Empirical—quantitative
Khan et al. [40]	2026	Australia; Melbourne	Migrant women with type 2 diabetes: 20	Diabetes, Depression	Ethnographic; life story narrative	Empirical—qualitative
Kohrt and Carruth [41] †	2022	Nepal; Ethiopia	Female ex-soldiers (*n* = 148); diabetes patients (*n* = 136)	Depression (with PTSD), Type 2 diabetes; Separate case studies	Ethnographic; mixed, semi-structured interviews	Empirical—qual./mixed
Lerman Ginzburg [38] †	2022	Puerto Rico (San Juan)	Men and women ≥ 18 y; *n* = 75	Obesity, Diabetes; Depression (in participants’ accounts)	Qualitative (semi-structured interviews)	Empirical—qual./mixed
McCurley et al. [10]	2019	USA	Latinos/Hispanics 18–74 y; *n* = 5247	Diabetes and Depression (comorbid)	Cohort (HCHS/SOL)	Empirical—quantitative
Mendenhall [13] ^B,C^	2016	USA (Chicago); India (Delhi); South Africa (Johannesburg)	Immigrants *n* = 121; diabetics *n* = 59; diabetics *n* = 27	Diabetes, Depression	Mixed—life-history narratives; Bt20 cohort	Empirical—qual./mixed
Mendenhall [34] ^C^	2014	South Africa (Johannesburg)	Black diabetic women; *n* = 27	Diabetes, Depression	Mixed (Bt20 cohort + in-depth narratives)	Empirical—qual./mixed
Mendenhall et al. [36] ^C^	2021	South Africa (Johannesburg)	Men and women; *n* = 783	Diabetes, Depression	Mixed (population-based surveys + in-depth interviews)	Empirical—qual./mixed
Mendenhall et al. [43]	2015	Kenya (Nairobi)	Men and women; *n* = 100	Syndemic suffering: Diabetes, Depression, HIV	Mixed—life-history narratives	Empirical—qual./mixed
Mendenhall and Norris [35] ^C^	2015	South Africa (Johannesburg)	Black diabetic women; *n* = 27	Diabetes, Depression, HIV, NCDs	Mixed (Bt20 cohort + in-depth interviews)	Empirical—qual./mixed
Page-Reeves et al. [44]	2019	USA (New Mexico)	Women 21–63 y; *n* = 21	Diabetes, Depression	Mixed (in-depth interviews + focus groups)	Empirical—qual./mixed
Saxena and Mendenhall [37] †	2022	India; China	Adults ≥ 50 y; *n* = 6090; *n* = 11,789	Hypertension, Type 2 diabetes, Functional disability; Depression (discussed, not analysed)	WHO SAGE	Empirical—quantitative
Weaver and Mendenhall [33] ^B^	2014	USA (Chicago); India (Delhi)	Migrants with diabetes *n* = 121; women *n* = 280	Diabetes, Depression	Case studies—medical anthropology	Empirical—qual./mixed
Zuñiga et al. [54] †	2022	USA (Alabama)	Adults ≥ 18 y; *n* = 5897	HIV, Diabetes, Treatment adherence, Depression, Quality of life, Self-management	Predictive and longitudinal national cohort	Empirical—quantitative
**Study Protocols (*n* = 2)**						
Awoke et al. [55]	2025	Ethiopia	Recently diagnosed diabetic adults; *n* = 485	Diabetes, Depression, Functional disability	Protocol—mixed, 6-month follow-up (questionnaires + phenomenological)	Study protocol (no results yet)
Friedman et al. [56] †	2024	USA	Adults with SSM ≥ 18 y; *n* = 1800	HIV/AIDS, NCDs (diabetes, hypertension, dyslipidaemia), Depression	Protocol—combined MACS/WIHS cohort (MWCCS)	Study protocol (no results yet)
**Editorials/Short Articles (*n* = 4)**						
Arena et al. [57]	2022	—	Children and young people with overweight/obesity	COVID-19, Depression, Overweight/Obesity, Diabetes	Editorial	Conceptual/non-empirical
Gallo et al. [58]	2021	—	—	Depression, Diabetes, Cognitive changes	Editorial	Conceptual/non-empirical
Mendenhall et al. [59]	2022	—	—	COVID-19, Diabetes, Depression, Musculoskeletal condition	Perspective	Conceptual/non-empirical
Ricceri F. [29]	2024	—	—	Multimorbidity among migrant populations; Diabetes, Depression (VIDDA syndemic)	Short article	Conceptual/non-empirical
**Books (*n* = 2)**						
Mendenhall [42]	2019	Multi-site (global)	Men and women in different parts of the world	Syndemic suffering, Diabetes, Depression	Mixed—ethnography (medical anthropology)	Empirical—qual./mixed (book)
Mendenhall [12] ^B^	2012	USA (Chicago)	Mexican women with diabetes; *n* = 121	Diabetes, Depression	Ethnography (life-history narratives)	Empirical—qual./mixed (book)
**Theses (*n* = 1)**						
Fox [60]	2016	USA (Arlington)	African-American men and women; *n* = 6	Diabetes, Depression, Syndemic suffering	Life-history narratives and experiences	Empirical—qual./mixed (thesis)

Note: —, field not applicable to this document type. Evidence type: “Empirical” = presents primary data; “Review (secondary)” = synthesises other studies; “Study protocol” = planned study, no results yet; “Conceptual/non-empirical” = editorial, perspective or commentary. Books and theses are classified by the evidence they contain. Shared study/dataset (superscript): A, Brazil National Health Survey 2013 [31,32]; B, Chicago/original VIDDA data [12,13,33]; C, Bt20 cohort, Johannesburg [13,34,35,36]—publications sharing a letter are not independent evidence. MSM, men who have sex with men; NCDs, non-communicable diseases; CHD, coronary heart disease; VACS, Veterans Aging Cohort Study; HCHS/SOL, Hispanic Community Health Study/Study of Latinos; SAGE, WHO Study on Global Ageing and Adult Health; Bt20, Birth to Twenty cohort; LCA, latent class analysis. † Met one core eligibility criterion indirectly (Section 2.3; Supplementary Table S1). Source: authors’ own elaboration.

Across 35 articles, we identified 160 authors, with each article featuring between 1 and 17 contributors. Mendenhall topped the list, participating in 34.28% of the publications, followed by Kohrt and Tsai, each contributing 8.57%. Meanwhile, Andersen, Bosire, Diderichsen, Lerman, Ndetei, and Norris appeared in 5.71% of articles each; the remaining 151 authors participated in only one article.

### 3.1. Analysed Comorbidities

The comorbidities studied and/or analysed alongside diabetes and depression refer to what Singer [11] defined as a bio–bio interface and included HIV/AIDS [35,50,51,52,54,56], overweight/obesity [38,45,57], non-communicable diseases—NCDs [35,37,47,56], COVID-19 [47,57,59], disability [31,37,55], anxiety [47,53], cognitive changes [58], mood disorders [53], musculoskeletal disorders [59], tuberculosis [49] and inflammatory markers [9]. In addition, some publications explicitly focused on studying or presenting the diabetes–depression dyad [10,12,13,32,33,34,36,39,41,44,52], including a few that also addressed syndemic suffering [42,43,60].

Few studies included in the present review emphasised the biological and/or pathophysiological pathways shared by the comorbidities studied; among them are Arena et al. [57], Byg et al. [50], Gallo et al. [58], Perez et al. [9], Saqib et al. [47] and Zúñiga et al. [54]. However, the bidirectional relationship between diabetes and depression has been widely studied and established in other studies [61,62,63], which have served as the basis for the present work. Researchers have strived to demonstrate that health conditions such as Non-Communicable Diseases (NCDs), including diabetes, and HIV, for example, create conditions for illnesses such as COVID-19 to worsen the burden of morbidity and mortality in mental health; however, information on how this occurs has not been expanded.

### 3.2. Social Factors Analysed in the Diabetes–Depression Syndemic

Following the operational definitions specified in Section 2.4, social factors were classified as structural or intermediary determinants according to the WHO SDH framework and the adapted VIDDA model. The synthesis below refers to the 35 included publications, regardless of study design, and is organised by the two domains of the VIDDA–WHO SDH adaptation. Table 2 maps each social factor identified in the literature onto the framework’s structural and intermediary determinants, showing where evidence concentrates and where it remains sparse. Overall, structural factors such as poverty, violence and migration were addressed more frequently than intermediary determinants such as material deprivation, psychosocial stressors and health-system barriers.

**Table 2:** Social Determinants of Health (WHO) and social factors identified in the included studies.

WHO Sub-Domain	Social Indicators/Factors Investigated	Studies (References)	Publications (*n*)	Of Which Empirical (*n*)
**Structural Social Determinants (WHO)**				
Socioeconomic and political context; governance; macroeconomic policies	Political violence; internal displacement; migration; political status/instability; HDI; structural violence; social inequality	[9,12,13,29,31,32,33,34,38,39,40,41,42,45,46,47,48,49,60]	19	13
Cultural norms and values	Symbolic violence/intersectional stigma: homophobia/transphobia; HIV-related stigma; gender-based violence/machismo	[9,10,12,13,29,33,37,38,42,46,47,48,49,50,52,54,56,58,59]	19	10
Social position; poverty	Stratification; poverty	[9,12,13,29,36,38,42,45,46,47,48,49,55,56,58,59,60]	17	7
Education	Educational level	[10,31,32,37,39,43,45,46,52,55,60]	11	9
Occupation	Underemployment/unemployment; work-related stress	[12,13,38,45,46,47,48]	7	4
Income	Financial stress; financial uncertainty/insecurity; household income	[9,10,12,13,32,33,35,36,37,38,39,42,43,45,46,47,50,55]	18	14
Sex	—	[13,30,31,35,38,41,44,50,54,60]	10	8
Race/ethnicity	—	[13,44,45,57]	4	1
**Intermediary Social Determinants (WHO)**				
Material circumstances	Material deprivation; food desert/food insecurity	[12,13,28,35,38,40,42,43,44,46,47,58]	12	8
Material circumstances (neighbourhood)	Neighbourhood: insecurity/violence; relationships with neighbours; environmental health; area (rural/urban)	[9,10,12,13,35,36,38,42,43,45,46,52,53,55]	14	11
Social cohesion	Social/institutional support (networks); religion; family/social conflicts; social isolation; marital status	[9,12,13,29,32,34,36,37,38,40,45,46,47,58]	14	9
Psychosocial factors	Interpersonal abuse: physical/psychological/sexual abuse; childhood traumatic history	[9,10,12,13,29,32,34,41,42,45,46,49,58]	13	8
Psychosocial factors (stress)	Stress: coping mechanisms/self-efficacy; illness-related distress; sexual-minority stress	[9,12,29,33,35,36,41,42,46,47,50]	11	7
Behavioural factors	Habits: alcohol/tobacco/psychoactive substance use; consumption of low-nutritional-value foods; physical inactivity/sedentary lifestyle	[9,13,29,32,35,46,50,51,52,55,56,57,59]	13	6
Biological factors	Functional impairment/disability; age/ageing	[29,32,45,46,47,52,55,58]	8	3
Health systems	Failures in healthcare; medical check-ups/illness management	[12,13,29,36,38,42,43,46,47,48,49,50,52,54,55]	15	9

Note. “Publications (*n*)” counts the distinct references listed in the row. “Of which empirical (*n*)” counts those that are empirical primary studies (as classified in Table 1); it excludes reviews refs. [9,46,47,48,49] and conceptual or not-yet-completed sources (editorials/perspectives and protocols: refs. [29,55,56,57,58,59]); books refs. [12,42] and the thesis ref. [60] are counted as empirical because they report primary data. Some references share a dataset and are not independent (Table 1: A [31,32]; B [12,13,33]; C [13,34,35,36]). HDI, Human Development Index. Source: authors’ own elaboration.

#### 3.2.1. Structural Social Determinants

Consistent with the structural domain outlined in Section 2.4, political violence, internal displacement, migration status, socioeconomic position and structural discrimination were grouped under “structural social determinants”. Most of the included publications examined at least one structural factor, with poverty and financial stress among the most frequently reported conditions. Because this scoping review did not appraise study quality and combined empirical with conceptual sources, these frequencies indicate how often factors were reported, not the strength or certainty of the underlying evidence. Several studies also highlighted social position through indicators such as education, occupation, income, and gender or ethnicity, often linking these dimensions to increased vulnerability to the diabetes–depression dyad and to syndemic clustering in specific populations and places.

Symbolic and structural forms of discrimination, such as intersectional stigma related to HIV status, gender, sexual orientation or race, were less consistently measured but emerged as important cross-cutting themes in ethnographic and mixed-methods work. These factors were typically described as reinforcing both depression and diabetes, for example, by shaping exposure to violence, precarious employment, and migration-related stress and by constraining access to stable housing and safe neighbourhoods. As shown in Table 2 and Table 3, these structural determinants were rarely modelled quantitatively as interaction terms, but they were central to qualitative narratives of syndemic suffering and social vulnerability.

#### 3.2.2. Intermediary Social Determinants

In line with the intermediary domain defined in Section 2.4, material circumstances, psychosocial stressors, behavioural mediators and health-system factors were coded as “intermediary social determinants”. Material deprivation, including food insecurity, overcrowded or unstable housing and neighbourhood insecurity, was documented in a smaller subset of the included publications, frequently in low-income urban settings or in studies focusing on migrants and marginalised communities. Neighbourhood conditions, such as perceived safety, relationships with neighbours, and environmental health, were also examined as potential contributors to stress, limited physical activity, and barriers to diabetes self-management.

Psychosocial stressors, such as interpersonal abuse, family conflict, social isolation and illness-related distress, appeared in a comparable number of publications and were often described as mediating the relationship between structural disadvantage and the diabetes–depression dyad. Behavioural and biological mediators (e.g., obesity, physical inactivity, substance use, functional disability) were more commonly treated as covariates or comorbidities than as explicitly social factors. Yet, several publications linked these mediators to social context, including gendered norms, caregiving roles and chronic stress. Health-system factors, such as fragmented mental-health services, limited access to specialist care and high out-of-pocket costs, were less frequently operationalised but were repeatedly highlighted in qualitative accounts as key barriers to the integrated management of diabetes and depression.

Taken together, these findings indicate that most included publications address social determinants of the diabetes–depression dyad but do so unevenly: structural factors are frequently recognised, intermediary factors are more variably documented, and the pathways connecting macro-level determinants, everyday circumstances, and biosocial interaction are rarely modelled formally. This pattern is consistent with the conceptual emphasis of the VIDDA–WHO SDH adaptation, while underscoring the need for future research to link structural and intermediary domains through explicit interaction and mediation frameworks.

### 3.3. Applications of Syndemic Interaction

In line with the secondary objective—to describe how biosocial interaction has been conceptualised and operationalised—this subsection summarises whether and how studies examined interaction between social factors and disease variables, either statistically (e.g., additive or multiplicative models, interaction terms, stratified analyses) or qualitatively through narratives of syndemic clustering and amplification. Drawing on the principles of syndemic theory proposed by Singer [64,65], this review emphasises interactions at the bio–bio and bio–social interfaces as a distinguishing feature of syndemics. The aim was therefore to determine how this principle has been introduced and tested in the studies included in this review.

Of the 35 included publications, 12 applied—or, in the case of one protocol, planned—quantitative statistical methods to examine the diabetes–depression dyad and its social determinants. To foreground the review’s central distinction between formal interaction and simple association, these studies are grouped below by whether they explicitly tested biosocial interaction. It is important to distinguish studies that explicitly modelled interaction, using additive or multiplicative terms as recommended by Tsai and Venkataramani [66] and Tsai et al. [67,68], from those that merely adjusted regression models for social covariates.

#### 3.3.1. Studies That Formally Tested Biosocial Interaction

Five of the 12 quantitative studies included an explicit interaction analysis. Two tested interaction on the additive scale: Diderichsen and Andersen [32] and Diderichsen et al. [31] applied generalised linear models specifically designed to assess additive (super-additive) interaction between diabetes, depression and social position, and both reported significant departures from additivity on disability (an excess of 20.0 percentage points in the comorbid, higher-educated group [31] as well as additive interactions of +10.8 percentage points in women and +21.2 in men [32]). Two tested interactions on the multiplicative scale: Mendenhall et al. [36] found a significant multimorbidity-by-stress product term (b = −0.16; 95% CI −0.27 to −0.05; *p* = 0.005), and Saxena and Mendenhall [37] reported significant multiplicative interactions (e.g., diabetes-by-hypertension on disability). One study, Eshak et al. [52], tested both additive and multiplicative interactions of substance abuse, depression and type 2 diabetes and found neither—an informative null result. Following Tsai and Venkataramani [66] and Tsai et al. [67,68], only these designs—interaction terms on a defined scale or purpose-built stratified analyses—constitute a formal test of the syndemic interaction principle.

#### 3.3.2. Studies That Adjusted for Social Covariates Without Testing Interaction

The remaining seven quantitative publications entered social determinants as covariates in association models without a formal interaction term: Byg et al. [50], in whom the small sample explicitly precluded interaction; Gupta et al. [53] and Dansero et al. [39], using Cox/latent-class hazard models; McCurley et al. [10] and Mendenhall et al. [43], using adjusted regression; and Page-Reeves et al. [44], using bivariate correlation. Awoke et al. [55] present a study protocol with no results yet. While informative about confounding and effect modification, these designs do not formally demonstrate the syndemic interaction principle in its strict sense. This pattern indicates that, although statistical models are widely used in the diabetes–depression literature, explicit testing of biosocial interaction remains relatively rare. In the VIDDA–WHO SDH adaptation, the literature populates the structural and intermediary domains unevenly and seldom connects them to the biosocial-interaction core—precisely the linkage the adaptation was designed to make visible.

#### 3.3.3. Qualitative and Mixed-Methods Evidence of Clustering and Amplification

Beyond formal statistical testing, a second way to apply Singer’s postulates is to qualitatively examine how social conditions magnify or attenuate health outcomes, particularly through ethnographic and in-depth narrative methods. Across the included publications, 22 of 35 (62.85%) reported syndemic clustering of the diabetes–depression dyad, often describing how structural and intermediary social determinants—such as violence, migration-related stress, poverty and interpersonal abuse—interact with biological vulnerability and daily self-management demands to produce mutually reinforcing cycles of illness and suffering (see Table 2). Here, interaction is documented through lived experience and contextualised trajectories rather than formal statistical terms, providing rich evidence of biosocial amplification even when quantitative interaction models are absent.

Taken together, the quantitative and qualitative findings show that most studies acknowledge interaction as a core feature of the diabetes–depression syndemic, but only five [31,32,36,37,52] formally operationalise it through additive or multiplicative modelling. Future research would benefit from combining the depth of ethnographic and narrative approaches with formal interaction frameworks, thereby strengthening the empirical demonstration of biosocial syndemic processes across diverse settings.

**Table 3:** Application of the syndemic interaction principle in the included studies.

Study	Statistical Method	Formal Interaction Test	Scale	How Interaction Was Handled/Main Result
Awoke et al. [55]	Poisson regression with robust variance estimator	No	NA	Study protocol (no results yet). Planned analysis: Poisson regression with robust variance for prevalence ratios; the protocol does not specify how interaction will be tested.
Byg et al. [50]	Bivariate and multivariable linear regression	No	NA	Interaction analysis explicitly precluded by the small sample (*n* = 88). Independent correlates of higher A1c (substance use +2.27; detectable HIV viral load +1.30; high triglycerides +0.92)—association/adjustment, not a formal interaction test.
Dansero et al. [39]	Latent class analysis (LCA); Cox proportional hazards	No	NA	Population-based cohort (Piedmont Longitudinal Study). LCA identified latent classes clustering T2DM, depression, education and place of birth; Cox linked classes to ER access and 5-year mortality. The authors apply the framework as an interpretive lens, “rather than as a formal test of biological or causal interaction.” High-comorbidity/low-education class had the worst outcomes (men: mortality HR 1.60; women: HR 1.43).
Diderichsen et al. [31]	Generalised linear models (binomial)	Yes	Additive	Formal additive-interaction test (departure from additivity). Positive (super-additive) diabetes × depression interaction on disability: observed comorbid disability exceeded the additive expectation by 20.0 pp (95% CI 11.6–28.5), rising to 33.8 in medium–low-HDI states.
Diderichsen and Andersen [32]	Generalised linear models (binomial)	Yes	Additive	Formal additive-interaction test. Diabetes × depression additive interaction on disability: women +10.8 pp (6.7–14.9), men +21.2 pp (15.9–26.6); education and obesity also interacted with the disorders.
Eshak et al. [52]	Logistic + linear binomial regression (logit link)	Yes	Additive and multiplicative	Formally tested additive and multiplicative interactions of substance abuse × depression × T2DM on retention in HIV care; none significant (neither additive nor multiplicative—no syndemic effect).
Gupta et al. [53]	Cox proportional hazards	No	NA	Cox associations (adjusted HRs); no interaction term. Higher mental-health service use in the most materially deprived (Q5 HR 1.07) and most residentially unstable (Q5 HR 1.13) neighbourhoods; no synergistic interaction demonstrated.
McCurley et al. [10]	Multinomial logistic regression	No	NA	Associations of social adversity with a four-level diabetes/depression outcome; no formal interaction term. Low income (OR 4.61), adverse childhood experiences, discrimination and neighbourhood problems linked to higher odds of comorbidity.
Mendenhall et al. [36]	Multivariable linear regression (product term)	Yes	Multiplicative	Formal product-term interaction: multimorbidity × stress significant—the negative association between multimorbidity and quality of life was amplified under high stress (b = −0.16; 95% CI −0.27 to −0.05; *p* = 0.005).
Mendenhall et al. [43]	Logistic regression + qualitative content analysis	No	NA	Logistic-regression associations plus qualitative analysis of “syndemic suffering”; no formal interaction term tested.
Page-Reeves et al. [44]	Spearman correlation (mixed-methods pilot)	No	NA	Pilot, community-engaged study; Spearman correlations between domains (rho ≈ 0.59); bivariate correlation not designed to assess interaction.
Saxena and Mendenhall [37]	Multivariable logistic regression (Firth penalised)	Yes	Multiplicative	Multiplicative interaction via product terms: significant gender × income interactions and a diabetes × hypertension interaction on disability (China OR 1.76, 95% CI 1.39–2.22; India OR 1.90, 1.38–2.63).

Note. NA, not applicable. “Formal interaction test” = explicit interaction term (additive or multiplicative) or a stratified analysis for biosocial synergy, as distinct from covariate adjustment. Biosocial interaction was formally tested in 5 of 12 studies: two additive (Diderichsen 2021, 2019 [31,32]; significant), two multiplicative (Mendenhall 2021 [36]; Saxena and Mendenhall 2022 [37]; significant), and one on both scales (Eshak 2024 [52]; null). pp, percentage points; HR, hazard ratio; OR, odds ratio; A1c, glycated haemoglobin. Source: authors’ own elaboration.

## 4. Discussion

We interpret these findings through the VIDDA–WHO SDH adaptation, which allows the fragmented evidence to be read as a coherent pathway running from structural determinants, through intermediary conditions, to the biosocial interaction at the core of the diabetes–depression syndemic. This scoping review mapped how social factors have been studied in relation to the diabetes–depression dyad within a syndemic framework and examined how biosocial interaction has been conceptualised and operationalised. Across the included literature, structural and intermediary determinants were commonly acknowledged, while their definitions and measurement were often implicit rather than explicit. Diabetes and depression were generally described as interrelated conditions, and integrated care was frequently proposed as a response to their co-occurrence. However, few studies formally assessed interaction or synergy; most described co-occurrence, accumulation of adversity, or reciprocal associations, without testing whether combined effects exceeded those expected from the individual conditions. This pattern resembles broader methodological critiques of syndemics research, which distinguish genuine interaction testing from additive counts of co-occurring conditions and caution against interpreting the latter as evidence of synergy [48,49].

Research and publications on the syndemic cluster of diabetes and depression began in 2012 with the book *Syndemic Suffering: Social Distress, Depression, and Diabetes among Mexican Immigrant Women* by Mendenhall, which included an ethnographic analysis of Mexican migrant women in Chicago (USA) [12]. These “life story narratives” provided the first evidence of the VIDDA syndemic and were followed by further publications through 2016. The studies, initially conducted in the USA, quickly spread to India, China, and some African countries, with qualitative research taking centre stage; Mendenhall and colleagues led all of them [13,33,34,35,43]. Subsequently, mixed-methods studies were introduced, incorporating secondary data from large-scale surveys and clinical records, alongside primary data from cohort analyses.

The first decade of research into the diabetes/depression dyad culminated in the publication of a second book and ushered in a new phase, with the incorporation of systematic reviews and growing interest in conducting studies in other countries such as Brazil [31,32], Mexico [45], Canada [53], and Puerto Rico [38]. The first theses on the subject appeared in 2016 [60], and some led to scientific publications [38,39].

Since 2022, there has been a surge in publications on the diabetes–depression dyad from a syndemic perspective, with greater heterogeneity in the types of studies or publications addressed, whilst qualitative studies—predominantly anthropological and ethnographic—continue to play a major role, with a select group of expert psychiatrists and medical anthropologists continuing to dominate the field. Nursing professionals were not involved in the publications included in this scoping review.

### 4.1. Evolving Evidence on the Diabetes–Depression Dyad and Multimorbidity

In what follows, evidence from the 35 included publications is explicitly distinguished from the external literature cited for context. The introduction documented robust evidence that diabetes and depression frequently co-occur, with meta-analyses showing approximately double the prevalence of depression in people with diabetes compared with those without, and longitudinal studies indicating bidirectional associations between depression, glycaemic control and complications [4,6]. The results of this review reinforce that picture: most empirical publications treat diabetes and depression as interlinked conditions, often embedded in broader multimorbidity clusters that include obesity, HIV, cardiovascular disease, disability and other non-communicable diseases. This reflects the syndemic premise that disease clusters arise in specific social, economic, and political contexts rather than as isolated clinical phenomena [19,69].

Beyond the included publications, recent burden studies and multimorbidity analyses underscore the magnitude of this dyad. Global Burden of Disease estimates show rising prevalence and DALYs for both diabetes and depressive disorders, with type 2 diabetes accounting for most diabetes-related DALYs and depressive disorders remaining leading causes of mental-health–related disability [8,20]. Also, beyond this review, population-based work in Mexico, Brazil and other middle-income settings has documented the high prevalence of concurrent type 2 diabetes and depressive symptoms, with strong associations with education, socioeconomic strata and neighbourhood disadvantage, reinforcing the argument that the dual burden is socially patterned [32,45]. Taken together, these findings support conceptualizing diabetes and depression not only as comorbid conditions but as components of a syndemic shaped by multimorbidity and social gradients in risk.

Placed in international context, the pattern mapped here aligns with recent syntheses conducted outside this review’s evidence base. A 2025 meta-analysis of 39 studies estimated that roughly one in three people with diabetes experience depression, with risk concentrated among socially disadvantaged groups such as those with unemployment or weak social support [1], echoing—on a far larger and more heterogeneous sample—the socially patterned clustering observed among the included publications. International intervention evidence, however, tempers optimistic readings of that clustering: a systematic review and meta-analysis of collaborative care for comorbid depression and diabetes found consistent improvements in depressive symptoms but no significant effect on glycaemic control [70]. This external evidence supports a cautious interpretation—the social patterning of the dyad is robust, whereas the clinical returns of acting on it remain uneven and context-dependent—rather than a claim of demonstrated synergy.

### 4.2. Structural and Intermediary Determinants: Progress and Gaps

By aligning the VIDDA framework with the WHO Social Determinants of Health model and defining structural vs. intermediary factors a priori, this review sought to move beyond illustrative diagrams toward a reproducible mapping of social domains. The main methodological contribution of this review is thus the VIDDA–WHO SDH adaptation itself: by defining structural and intermediary domains a priori and linking them to biosocial interaction, it converts VIDDA from a context-specific, largely descriptive model into a reproducible charting and interpretive framework applicable across settings. The results show that most empirical publications address structural determinants such as poverty, structural violence, migration status, and social position (education, occupation, income, gender, ethnicity), with over half of the empirical studies explicitly analysing at least one structural factor. Ethnographic and mixed-methods work—particularly studies inspired by Singer and Mendenhall—highlighted how these determinants are experienced as chronic insecurity, discrimination, and constrained opportunities, thereby shaping both exposure to risk and the lived experience of diabetes and depression [12,19].

Intermediary determinants were also present but were measured more variably. Material deprivation (food insecurity, unstable or overcrowded housing, unsafe neighbourhoods) and psychosocial stressors (interpersonal abuse, family conflict, isolation, distress) appeared in roughly one-third to two-thirds of empirical publications, often in studies focusing on migrants, low-income urban populations, or ethnic minority communities. Health-system factors, such as fragmented mental-health services, limited access to specialist care, and high out-of-pocket costs, were less frequently operationalised quantitatively but were repeatedly described qualitatively as barriers to integrated management of diabetes and depression [10,16]. This pattern mirrors broader evidence that social determinants operate at multiple levels and that material and psychosocial conditions are key pathways through which structural disadvantage affects multimorbidity and distress [48,69].

A notable gap is that few studies explicitly link structural and intermediary domains through formal mediation or pathway models. Most treat these domains as parallel covariates or contextual descriptors rather than as components of a causal chain connecting macro-level conditions, everyday circumstances, mental health and metabolic outcomes. Future research would benefit from designs that explicitly test how structural determinants (e.g., income, education, discrimination) operate through material hardship, psychosocial stress and care access to shape the diabetes–depression syndemic [69].

### 4.3. Biosocial Interaction: Conceptual Adoption vs. Formal Testing

A key finding of this review is the discrepancy between how often interaction is invoked conceptually and how rarely it is tested statistically. In our sample, 12 of the 35 included publications used regression or related techniques to examine associations between clinical variables and social factors, but only a minority included explicit additive or multiplicative interaction terms or stratified analyses designed to test biosocial interaction in the syndemic sense. This is consistent with methodological notes by Tsai and Venkataramani, who caution that simply adjusting for multiple “syndemic” conditions or summing risk scores does not demonstrate synergy; instead, formal interaction (e.g., relative excess risk due to interaction, interaction terms on additive or multiplicative scales) is needed to assess whether combined effects exceed what would be expected from each factor alone.

The work by Diderichsen and colleagues [31,32], which applied generalised linear models to test additive interaction between clinical and social variables using national survey data, illustrates what more formal biosocial modelling can look like in practice. However, most quantitative publications in this review included social determinants as covariates in adjusted models without testing interaction terms. While such models inform associations and confounding, they stop short of demonstrating the “synergistic interaction” that syndemic theory posits as a core tenet. This aligns with recent editorial commentary noting that the syndemics literature has struggled to address the second tenet—synergy—empirically.

Qualitative and mixed-methods studies provide complementary evidence, documenting how structural and intermediary determinants amplify illness trajectories and treatment burdens. Ethnographic work within the VIDDA framework and related studies describe how violence, migration-related isolation, poverty, and abuse intersect with diabetes and depression in specific contexts, producing cycles of suffering that are experienced as more than the sum of individual conditions [12,13,33,34,35,38,42,43]. In these publications, interaction is evident in the narratives and life histories even when it is not expressed in statistical terms, reinforcing the value of qualitative approaches for capturing biosocial processes.

Taken together, the findings suggest that interaction is widely recognised conceptually but under-tested statistically. Future syndemics research on diabetes and depression should integrate formal interaction approaches, such as those proposed by Tsai and colleagues [66,68,71], with qualitative and mixed-methods designs, thereby strengthening both the theoretical and empirical foundations of biosocial synergy.

### 4.4. Implications for Integrated Care and Policy

The updated introduction and the findings of this review converge on the need for integrated, patient-centred care that addresses both diabetes and depression alongside their social determinants. Systematic reviews of collaborative and stepped-care models indicate that integrating mental health into diabetes services improves depressive outcomes and diabetes-specific distress and may yield modest improvements in HbA1c, although effects on glycaemic control are not uniform across interventions. Technology-supported psychological interventions, including eHealth programs, appear effective in reducing depressive symptoms, but their impact on glycaemic control is more limited and heterogeneous [16].

Our synthesis of social factors reinforces that those structural and intermediary determinants, such as income, education, neighbourhood disadvantage, discrimination, material hardship and health-system fragmentation, shape both exposure to the diabetes–depression dyad and the feasibility of integrated care. Among the included studies, those of U.S. Latinos [10], for example, show that low household income, adverse childhood experiences, ethnic discrimination and neighbourhood problems are strongly associated with diabetes/depression comorbidity and suggest that interventions at both individual and neighbourhood levels, including integrated care in community clinics and improvements in local safety and food environments, may reduce syndemic-related inequities [10].

These findings imply that integrated care models will be most effective when they explicitly incorporate screening and support for social determinants—such as food insecurity, housing instability, interpersonal violence and discrimination—rather than focusing solely on pharmacologic management and brief psychological interventions. Syndemic thinking can help design such models by highlighting the need to address clustering and interaction of clinical conditions and social adversities, and by encouraging partnership across health, social services and community sectors [72,73].

### 4.5. Conceptual and Methodological Considerations for Future Research

Finally, this review speaks to ongoing debates about how to conceptualise and study syndemics. Recent critiques warn that broad claims of “mutually reinforcing factors” without clear interaction tests risk stretching the concept to the point of ambiguity. Our findings support a balanced approach: syndemic theory remains useful for organizing thinking about clustered diseases and social determinants, particularly in contexts of inequality, but its empirical implementation requires clearer operational definitions, attention to scale (additive vs. multiplicative), and explicit distinction between co-occurrence, correlation, and synergy.

By specifying in advance which factors were treated as structural vs. intermediary, including both empirical and conceptual work, and separating statistical interaction from simple adjustment for covariates, this review aimed to make the VIDDA–WHO adaptation reproducible rather than merely illustrative. Future work could extend this approach through the following:

- Incorporating longitudinal designs that track changes in structural and intermediary determinants alongside clinical trajectories;
- Using formal interaction and mediation models to test pathways from structural disadvantage through intermediary conditions to multimorbidity and distress;
- Integrating spatial epidemiology and neighbourhood-level measures to examine clustering and context;
- Combining these quantitative approaches with ethnographic and participatory methods that capture the lived experiences of syndemic suffering.

Such designs would help clarify when and how diabetes and depression function as a syndemic in different populations and what combinations of clinical, psychosocial, and structural interventions are most effective for reducing the dual burden.

### 4.6. Implications for Nursing Practice and Research

Because the diabetes–depression syndemic is shaped by structural and intermediary determinants rather than by biology alone, nurses—who provide continuous, longitudinal contact across primary, community and hospital settings—are strategically positioned to identify and respond to it and may have an important role in identifying and addressing these interacting clinical and social needs. This review’s findings suggest several priorities for nursing practice. These priorities are derived from the conceptual VIDDA–WHO synthesis and the social determinants mapped here; because the included studies rarely evaluated nursing-led interventions, read them as evidence-informed proposals rather than effects directly demonstrated by the reviewed evidence.

Specifically, the contribution of this review to nursing knowledge lies in reframing the co-occurrence of diabetes and depression as inseparable from the social and contextual circumstances that shape its onset, interaction and management. Synthesising the evidence on the social factors surrounding the dyad through a syndemic lens gives nurses a structured way not only to identify individual vulnerabilities but also to articulate health and social services as more culturally safe and contextualised care. This interpretive contribution comes from the mapped evidence rather than from nursing interventions evaluated in the included studies; accordingly, any potential to reduce health inequities remains a hypothesis to be confirmed through nurse-led causal and interventional research, as outlined below.

First, routine nursing assessment should extend beyond glycaemic and mood parameters to incorporate structured screening for the social determinants most consistently identified here, including food and housing insecurity, exposure to interpersonal and structural violence, migration-related stress, and financial hardship, alongside validated screening for depressive symptoms and diabetes-related distress. Embedding such screening in nursing consultations could support earlier identification of individuals whose clinical trajectory is being amplified by social adversity. Systematic-review evidence indicates that linking patients to community-based social support (social prescribing) can improve quality of life and disease-specific psychological outcomes in long-term conditions, although effects on metabolic markers such as HbA1c remain small and inconsistent [74]; screening is therefore best framed as a route to psychosocial support rather than a guaranteed metabolic intervention.

Second, the fragmentation of medical and mental-health services repeatedly reported in the included studies points to a coordinating role for nursing. Nurses, particularly advanced-practice and community/public-health nurses, are well placed to lead case management and intersectoral coordination—linking primary care with mental-health, social-protection, and community services, ensuring timely referral, and sustaining continuity of care for people managing two interacting chronic conditions. This is consistent with trial evidence that collaborative, integrated models reliably improve depressive outcomes in people with comorbid diabetes and depression, even where glycaemic gains are not observed [70].

Third, given the prominence of stigma, self-devaluation and diminished autonomy in the reviewed literature, interventions should be delivered through a culturally safe, person-centred and trauma-informed lens. Nurse-led education, peer-support facilitation and self-management support can strengthen coping resources and mitigate the isolation and shame that reinforce the syndemic cycle, particularly among migrant, ethnic-minority and low-income communities. Although nursing cultural-competence interventions show promising effects on providers’ competence, their impact on patient outcomes is still uncertain and under-evaluated [75], underscoring the need to test culturally safe nursing care against clinical and equity endpoints.

Fourth, at the system and policy level, these findings support integrating social-needs screening and syndemic-informed pathways into diabetes and mental-health care standards, although further evaluation is needed to assess the effectiveness of such approaches. Continuing (permanent) education is needed to equip the nursing workforce to recognise clustering and biosocial interaction and to act on social determinants rather than on isolated diseases.

From a research standpoint, the scarcity of studies formally testing biosocial interaction and the near-absence of nursing-led or nursing-focused investigations represent key gaps. Future nursing science should use longitudinal and interventional designs—including nurse-delivered, community-based, and integrated-care models—that explicitly link structural determinants, intermediary conditions, and clinical outcomes and evaluate whether nursing interventions can attenuate the combined burden of diabetes and depression in marginalised populations.

#### 4.6.1. Limitations

A notable methodological limitation is the heterogeneity in how the included studies conceptualised and operationalised structural and intermediary social determinants, which constrains the identification of consistent syndemic patterns and limits cross-study comparability. In several publications, factors that align with structural determinants (e.g., poverty, discrimination, migration regimes) were treated interchangeably with intermediary conditions (e.g., material deprivation, psychosocial stress, care access), making it difficult to trace pathways from macro-level contexts to proximal experiences and outcomes.

Furthermore, the predominance of cross-sectional and retrospective designs in the included empirical studies restricts causal inference regarding the directionality of interactions between diabetes and depression within adverse social contexts. Without longitudinal data, it remains challenging to disentangle whether social conditions primarily increase the risk of syndemic clustering, worsen trajectories once the dyad is established, or both. These limitations highlight the need for longitudinal, integrative studies that combine quantitative and qualitative methodologies to capture both structural-level dynamics—political, economic and cultural—and their proximal effects on individual and population health.

Another limitation is potential publication bias, as the review did not search trial registries or systematically contact authors for unpublished data. The use of three core search terms (“syndemic”, “diabetes”, “depression”) may also have excluded studies that conceptually align with syndemic theory but do not use the term explicitly, potentially underestimating the relevant evidence base. Including the grey and non-peer-reviewed literature, while necessary to capture the conceptual foundations of syndemic research, introduces heterogeneity in methodological rigour that we could not formally appraise, consistent with scoping-review methodology; to mitigate this, empirical and conceptual sources were separated in all quantitative summaries, and findings drawn from ethnographic and theoretical works should be read as conceptual rather than confirmatory evidence.

Most importantly, three features of the evidence base impose a hard boundary on interpretation. First, mapping the heterogeneous terminology of the original publications onto the VIDDA–WHO categories inevitably involved interpretative judgement, introducing some subjectivity in classification. Second, several included publications derive from the same underlying study or dataset (marked A–C in Table 1 and Table 2) and, although counted as distinct publications, do not constitute independent empirical evidence. Third, and decisively, we undertook no formal critical appraisal. Taken together, this means that every frequency-based descriptor in this review—including “predominant”, “most frequently reported” and “consistently identified”—denotes only how often a factor appeared across the included publications, not the magnitude, direction, or certainty of its association with the diabetes–depression dyad: a determinant may be reported frequently yet rest on weak or non-peer-reviewed evidence, while one reported only once may be supported by a large, well-designed study, and shared datasets may further inflate apparent frequencies. Consequently, the review maps the breadth and nature of the evidence. Still, it cannot establish strength or certainty, and the clinical and nursing implications in Section 4.6 should be read as hypothesis-generating and framework-informed rather than as evidence-based recommendations of demonstrated effectiveness; confirming whether acting on these determinants improves outcomes or reduces inequities will require appraised, longitudinal, and interventional research. In addition, seven of the 35 included publications met one of the two core eligibility criteria only indirectly (Supplementary Table S1). Their inclusion broadens the map, but not every included source addresses the diabetes–depression dyad as its primary focus.

Finally, we did not consult nurses, patients, or community stakeholders; while not required for a scoping review, such involvement could have helped contextualise how to translate the mapped evidence into nursing practice recommendations.

#### 4.6.2. Strengths

To our knowledge, this is one of the first scoping reviews to systematically map the social interaction dimension of the diabetes–depression syndemic, addressing a critical gap in the syndemic theory literature. By centring the analysis on how structural and intermediary social determinants generate adverse biological and psychosocial synergies that amplify disease burden, this study offers a theoretically grounded contribution to understanding how social contexts potentiate the co-occurrence and mutual reinforcement of diabetes and depression.

Another strength lies in the explicit, a priori alignment of the VIDDA framework with the WHO Social Determinants of Health model and the operational distinction between structural and intermediary domains, which enhances reproducibility and clarity compared with purely illustrative adaptations. The breadth of the scoping review methodology, encompassing 35 publications across diverse geographic and sociocultural contexts and including quantitative, qualitative, and conceptual work, further strengthens the scope and transferability of the findings while allowing identification of important gaps in interaction modelling and integrated care.

## 5. Conclusions

Over the past 14 years, a wide but unevenly distributed range of structural and intermediary social determinants has been examined in relation to the diabetes–depression syndemic, with coverage varying considerably across studies and geographic contexts. Beyond mapping this evidence, the review offers the VIDDA–WHO SDH adaptation as a reproducible framework that links structural and intermediary determinants to the biosocial core of the dyad, giving researchers and nurses a structured lens to identify and interpret its social patterning. Within this map, structural determinants such as violence, migration, stigma and economic disadvantage, together with intermediary determinants such as access to health services, neighbourhood conditions, social cohesion and interpersonal abuse, were the most frequently reported—an indication of how often each was studied rather than of its relative causal weight.

The evidence indicates that social and economic conditions are closely associated with the co-occurrence of diabetes and depression, particularly in contexts of inequality; however, it does not establish that these conditions amplify one another, since few studies have formally tested biosocial interaction. The conceptual usefulness of the syndemic framework should therefore be distinguished from the current strength of empirical evidence for true biosocial synergy, which remains limited across populations and settings. Shared pathophysiological pathways—dysregulated stress responses, inflammation and metabolic disturbance—are consistent with an interaction between the two conditions within an adverse social context, but confirming and quantifying that interaction remains an open empirical question.

Taken together, these findings support syndemic thinking as an organising lens for equity-oriented research and nursing practice while cautioning against generalising the form or intensity of the diabetes–depression syndemic beyond the settings in which it has been studied. The differences observed across countries and regions reinforce the value of contextualised analysis and point to appraised, longitudinal and interventional research—including nurse-led and integrated-care designs—as the necessary next step for testing whether acting on social determinants improves outcomes.

## Figures and Tables

**Figure 1:**
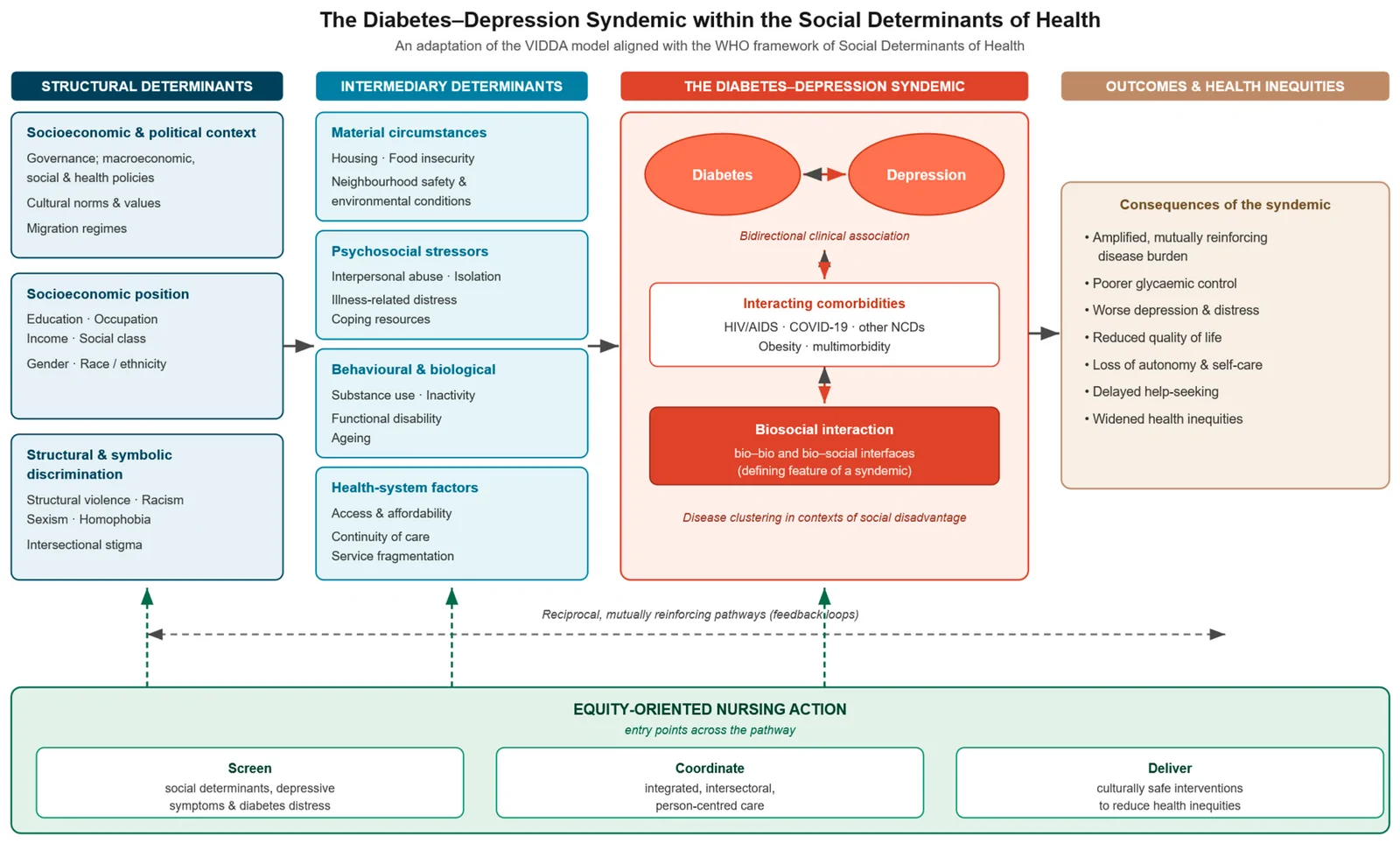
Conceptual framework of the diabetes–depression syndemic within the social determinants of health, adapting the VIDDA model to the WHO framework. Structural determinants operate through intermediary determinants to shape the diabetes–depression syndemic and its interacting comorbidities; biosocial interaction (bio–bio and bio–social interfaces) is the defining feature, and double-headed arrows denote reciprocal, mutually reinforcing pathways. The lower band indicates entry points for equity-oriented nursing action (screening, care coordination, culturally safe interventions). SDs, social determinants; NCDs, non-communicable diseases; SDHs, social determinants of health. Adapted from the WHO framework of social determinants of health [21] and the VIDDA model [13].

**Figure 2:**
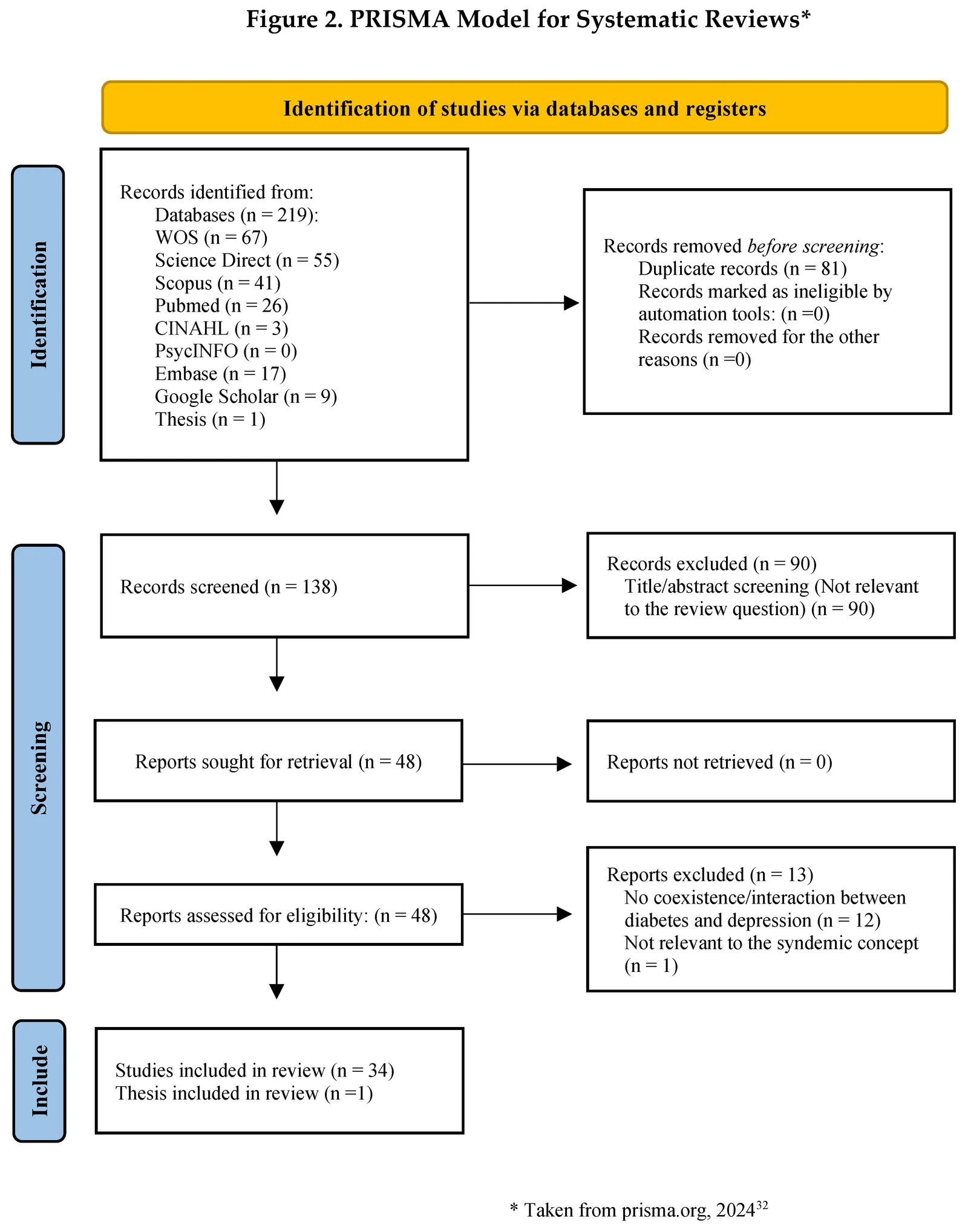
PRISMA 2020 flow diagram for scoping reviews/PRISMA-ScR [30].

## Data Availability

No new data were created or analysed in this study. All data supporting the findings are available within the article and its Supplementary Materials.

## Supplementary Materials

The following supporting information can be downloaded at: https://www.mdpi.com/article/10.3390/nursrep16100360/s1. Search strategy—complete search strings for all nine sources (PubMed, Web of Science, Scopus, ScienceDirect, CINAHL, PsycINFO, Embase, Google Scholar, and thesis registers), including platform, cut-off date (20 September 2026), filters applied, and a summary table of records retrieved per source (total *n* = 219). File S1: Search strategy; File S2: PRISMA-ScR checklist; File S3: Eligibility audit.

## Public Involvement Statement

There was no public involvement in any aspect of this research.

## Guidelines and Standards Statement

This scoping review was reported in accordance with the PRISMA extension for Scoping Reviews (PRISMA-ScR) checklist and was conducted following the Joanna Briggs Institute (JBI) methodology for scoping reviews.

## Abbreviations

The following abbreviations are used in this manuscript:

HIV	Human immunodeficiency virus
AIDS	Acquired immunodeficiency syndrome
VIDDA	Structural violence, immigration, diabetes, depression, interpersonal abuse
HPA	Hypothalamic-Pituitary-Adrenal
GBD	Global Burden of Disease Study
DALY	Disability-Adjusted Life Years
WHO	World Health Organization
SDH	Social Determinants of Health
NCDs	Non-communicable diseases
PCC	Population, Concept, and Context
JBI	Joanna Briggs Institute
