# Supplementary material for: Social Determinants of the Diabetes–Depression Dyad Within a Syndemic Framework: A Scoping Review
Source: Nurs Rep. 2026 Oct 12;16(10):360. doi: 10.3390/nursrep16100360 (PMC13708043; doi:10.3390/nursrep16100360)
Supplement: Supplementary file 1 [file nursrep-16-00360-s001.zip › S1 Search strategy.pdf]

Supplementary material - Search strategy

1. PubMed (Cut-off date: 20/09/2026)
((("Syndemics"[MeSH Terms] OR "Syndemic"[tiab] OR "Syndemic theory"[tiab]
OR "Syndemic framework"[tiab] OR "Syndemics"[tiab])
AND
("Diabetes Mellitus"[MeSH Terms] OR "Diabetes Mellitus, Type 2"[MeSH Terms]
OR "Diabetes"[tiab] OR "Type 2 diabetes"[tiab] OR "T2DM"[tiab])
AND
("Depression"[MeSH Terms] OR "Depressive Disorder"[MeSH Terms]
OR "Depressive Disorder, Major"[MeSH Terms] OR "Depression"[tiab]
OR "Depressive symptoms"[tiab] OR "Depressive disorder"[tiab]
OR "Mental health"[tiab])))
Filters applied: none (no language, date, or design restrictions)

2. Scopus (Cut-off date: 20/09/2026)
TITLE-ABS-KEY (
("syndemic" OR "syndemics" OR "syndemic theory" OR "syndemic framework")
AND
("diabetes" OR "diabetes mellitus" OR "type 2 diabetes" OR "T2DM")
AND
("depression" OR "depressive disorder" OR "depressive symptoms"
OR "major depressive disorder")
)
Filters applied: none

3. Web of Science — WOS (Cut-off date: 20/09/2026)
TS = ("syndemic" OR "syndemics" OR "syndemic theory" OR "syndemic framework")
AND
TS = ("diabetes" OR "diabetes mellitus" OR "type 2 diabetes")
AND
TS = ("depression" OR "depressive disorder" OR "depressive symptoms")
Filters applied: none
Collections: Web of Science Core Collection (SCI-EXPANDED, SSCI, A&HCI, ESCI)

4. Science Direct (Cut-off date: 20/09/2026)
title-abstr-key("syndemic" OR "syndemics" OR "syndemic theory")
AND
title-abstr-key("diabetes" OR "diabetes mellitus")
AND
title-abstr-key("depression" OR "depressive disorder" OR "depressive symptoms")
Filters applied: none

5. Google Scholar (advanced search) - Grey literature (Cut-off date: 20/09/2026)
In **every** word - In the article title: "syndemic" AND "diabetes" AND "depression"
Filters applied: Do not include citations

6. CINAHL (Cut-off date: 20/09/2026)
("syndemic" OR "syndemics" OR "syndemic theory" OR "syndemic framework")
AND ("diabetes" OR "diabetes mellitus" OR "type 2 diabetes" OR "T2DM")
AND ("depression" OR "depressive disorder" OR "depressive symptoms" OR "major depressive disorder")
Filters applied: none

7. PsycINFO (Cut-off date: 20/09/2026)
("syndemic" OR "syndemics" OR "syndemic theory" OR "syndemic framework")
AND ("diabetes" OR "diabetes mellitus" OR "type 2 diabetes" OR "T2DM")
AND ("depression" OR "depressive disorder" OR "depressive symptoms" OR "major depressive disorder")
Filters applied: none

8. EMBASE (Cut-off date: 20/09/2026)
("syndemic" OR "syndemics" OR "syndemic theory" OR "syndemic framework")
AND ("diabetes" OR "diabetes mellitus" OR "type 2 diabetes" OR "T2DM")
AND ("depression" OR "depressive disorder" OR "depressive symptoms" OR "major depressive disorder")
Filters applied: none

9. Theses — ProQuest (Cut-off date: 20/09/2026)
Search using the terms "syndemic", "diabetes" and "depression".

Records retrieved: 1

Database	Platform	Cut-off date	Summary Strategy	n
PubMed	NLM	20/09/2026	MeSH + tiab; no filters	26
Web of Science	Clarivate	20/09/2026	TS; no filters	67
Scopus	Elsevier	20/09/2026	TITLE-ABS-KEY; no filters	41
ScienceDirect	Elsevier	20/09/2026	title-abstr-key; no filters	55
CINAHL	EBSCOhost	20/09/2026	Database search; no filters	3
PsycINFO	EBSCOhost	20/09/2026	Database search; no filters	0
EMBASE	Elsevier	20/09/2026	Database search; no filters	17
Google Scholar	Google	20/09/2026	Free search; grey literature	9
Theses	ProQuest	20/09/2026	Thesis search	1
Total				219
