# Supplementary material for: Social Determinants of the Diabetes–Depression Dyad Within a Syndemic Framework: A Scoping Review
Source: Nurs Rep. 2026 Oct 12;16(10):360. doi: 10.3390/nursrep16100360 (PMC13708043; doi:10.3390/nursrep16100360)
Supplement: Supplementary file 1 [file nursrep-16-00360-s001.zip › S3_Eligibility_Audit.pdf]

Supplementary Table S1. Eligibility audit of the 35 included publications and of the 13 reports excluded at full text

Two reviewers (D.P.L.B. and B.Y.H.S.) independently assessed the full text of every publication against the two core eligibility criteria stated in Section 2.3 of the article: criterion 2, explicit reference to the coexistence of diabetes and depression; and criterion 3, explicit use of a syndemic theoretical framework (e.g., "syndemic", "VIDDA", biosocial interaction). Titles, abstracts and indexing terms alone were not used to decide eligibility. Each criterion was recorded as met directly or indirectly:

- Criterion 2, direct: the publication explicitly addresses diabetes and depression as coexisting or interacting conditions, as its main focus or as one of the disease clusters it analyses or reviews.
- Criterion 2, indirect: the publication addresses both conditions, but their coexistence is not the subject of explicit analysis. For example, one of them is a component of a wider multimorbidity cluster or analytical model, an adjustment variable, or is discussed as part of the syndemic context without being analysed; or the two conditions are examined in separate case studies.
- Criterion 3, direct: syndemic theory is the stated conceptual or analytical framework.
- Criterion 3, indirect: syndemic concepts are used to frame or interpret the findings without structuring the analysis.

Result. All 35 included publications met both criteria at one of the two levels. Twenty-eight met both directly. Seven, marked †, met one criterion indirectly: five for criterion 2 and two for criterion 3. These seven were retained because they show how the dyad appears within broader syndemic research, and they are treated in the article as contextual, not core, evidence.

Part A. Included publications (n = 35)

Study [reference]	Year	Conditions examined	Criterion 2: diabetes–depression coexistence	Criterion 3: syndemic framework	Basis for the decision (supporting passage and page where eligibility was questioned)
Review studies (n = 5)					
Gizamba et al. [44]	2023	Multimorbidity (review of syndemics research)	Direct	Direct	Review of place-related contextual factors in syndemics research; the diabetes–depression syndemic is among the syndemics mapped.
Mendenhall et al. [47]	2017	Diabetes, HIV, Depression, TB	Direct	Direct	Title names both conditions and the framework: "Non-communicable disease syndemics: poverty, depression, and diabetes among low-income populations".
Perez et al. [9]	2024	Diabetes, Depression, Inflammatory markers	Direct	Direct	Scoping review of the social determinants of inflammatory markers "linking depression and type 2 diabetes" (title); reviewed within a syndemic framing.
Saqib et al. [45]	2023	COVID-19, Anxiety, Depression, Diabetes, Hypertension, Asthma, CHD	Direct	Direct	Title names the framework ("A Syndemic Perspective"); depression and diabetes are among the conditions reviewed together.
Singer et al. [46]	2020	Multimorbidity (review of syndemics research)	Direct	Direct	Review of syndemics research 2015–2019; explicit reference to diabetes–depression coexistence among the syndemics reviewed.
Original articles (n = 21)					
Agudelo-Botero et al. [41]	2022	Type 2 diabetes, Depression, Obesity	Direct	Direct	Title names both conditions and the framework: "Type 2 diabetes and depressive symptoms in the adult population in Mexico: a syndemic approach…".
Byg et al. [48]	2016	Diabetes, HIV, Hypertension, Depression, Substance abuse	Direct	Direct	Syndemic framework named in the title. Depression is one of the co-occurring conditions analysed in men with HIV and type 2 diabetes: "more men with inadequate glycemic control (A1c > 7.5 %) had … depression (67 vs. 39 %, p = 0.018)" (abstract; results, pp. 5–6).

Study [reference]	Year	Conditions examined	Criterion 2: diabetes–depression coexistence	Criterion 3: syndemic framework	Basis for the decision (supporting passage and page where eligibility was questioned)
Chichetto et al. [49] †	2019	Depressive symptoms, Unhealthy alcohol use, Cigarette use, HIV; all-cause mortality; Diabetes (adjustment variable)	Indirect	Direct	Criterion 3: the exposure is a syndemic of unhealthy alcohol use, cigarette use and depressive symptoms (title; p. 1). Criterion 2: diabetes is not part of the syndemic and is not an outcome; the outcome is all-cause mortality. Diabetes is defined as a covariate ("Diabetes was defined as having any of the following indicators: abnormal glucose measurement, use of medications to treat diabetes, or at least 1 inpatient or 2 outpatient … codes for diabetes", Methods, p. 2), reported as a baseline characteristic by number of syndemic conditions (Table 1, p. 4) and entered as an adjustment variable in the mortality models (Table 3, p. 6).
Dansero et al. [43]	2026	Diabetes, Depression	Direct	Direct	Title names both conditions and the framework: "Syndemic interactions in northwest Italy: education, place of birth, and the co-occurrence of type 2 diabetes and depression".
Diderichsen et al. [30] (A)	2021	Disability, Diabetes, Depression	Direct	Direct	Title names both conditions and the framework: "Depression and diabetes: The role of syndemics in the social inequality of disability". Shared dataset A.
Diderichsen & Andersen [31] (A)	2019	Diabetes, Depression	Direct	Direct	Title names both conditions and the framework: "The syndemics of diabetes and depression in Brazil – An epidemiological analysis". Shared dataset A.
Eshak et al. [50]	2024	Depression, Diabetes, HIV, HIV treatment retention	Direct	Direct	Title names both conditions and the framework: "Syndemic of Substance Abuse, Depression, and Type 2 Diabetes and Their Association with Retention in Care Among People Living with HIV".
Gupta et al. [51] †	2022	Diabetes, Depression, Mood disorders, Anxiety	Direct	Indirect	Criterion 2: cohort of adults with diabetes; the outcome is "use of medical or hospital services at least once in a given year for a mood disorder (e.g., depression, neurosis, affective psychosis), an anxiety disorder … or both" (p. 2). Criterion 3: syndemic concepts frame the rationale and the interpretation without structuring the analysis. The introduction refers to "syndemic clusterings of comorbid diabetes and mental illness within and across populations" (p. 2), and the conclusion to "the syndemics of neighbourhood socioenvironments and mental health implications in populations with high diabetes burden" (p. 4). "Syndemic" is a keyword (p. 1).
Khan et al. [38]	2026	Diabetes, Depression	Direct	Direct	Title names the framework and the conditions: "Storying syndemics: understanding migrant women's experiences of diabetes and distress through narratives of self-care".
Kohrt & Carruth [36] †	2022	Depression (with PTSD), Type 2 diabetes; separate case studies	Indirect	Direct	Criterion 3: syndemic theory is the stated framework (title; abstract, p. 1). Criterion 2: the two conditions are examined in separate case studies, "posttraumatic stress disorder (PTSD) and depression co-morbidity among female former child soldiers" in Nepal and "the high rates of type 2 diabetes" among Somalis in Ethiopia (abstract, p. 1). Their coexistence is referred to but not analysed: the VIDDA model "has been used to understand why Mexican immigrants are more likely to have greater co-morbidity of depression and diabetes" (p. 2), and "stress, trauma, clinical depression, and violence play a causal role in the diagnosis and experience of diabetes" (p. 7).

Study [reference]	Year	Conditions examined	Criterion 2: diabetes–depression coexistence	Criterion 3: syndemic framework	Basis for the decision (supporting passage and page where eligibility was questioned)
Lerman Ginzburg [52] †	2022	Obesity, Diabetes; Depression (in participants' accounts)	Indirect	Direct	Criterion 3: syndemic theory is the stated framework (title). Criterion 2: depression is not a studied condition. It appears in the framing, where obesity "is a risk factor for illnesses such as diabetes and depression" (p. 1), and in participants' accounts: "the most common explanation was that people became depressed as a result of the high unemployment and crime rates, ate large quantities of cheap, high-fat food to cope with their problems, developed obesity as a result, and thence diabetes" (p. 6).
McCurley et al. [10]	2019	Diabetes & Depression (comorbid)	Direct	Direct	Title names comorbid diabetes and depression; associations with social adversity analysed within a syndemic framework.
Mendenhall [13] (B, C)	2016	Diabetes, Depression	Direct	Direct	Title names both conditions and the framework: "Beyond Comorbidity: A Critical Perspective of Syndemic Depression and Diabetes in Cross-cultural Contexts". Shared datasets B and C.
Mendenhall [33] (C)	2014	Diabetes, Depression	Direct	Direct	Framework named in the title ("Syndemic Suffering in Soweto…"); diabetes and depression analysed together in women with diabetes. Shared dataset C.
Mendenhall et al. [35] (C)	2021	Diabetes, Depression	Direct	Direct	Framework named in the title ("A mixed-methods, population-based study of a syndemic in Soweto, South Africa"); diabetes and depression analysed together. Shared dataset C.
Mendenhall et al. [39]	2015	Syndemic suffering: Diabetes, Depression, HIV	Direct	Direct	Title names diabetes and the framework ("Stress, diabetes, and infection: Syndemic suffering at an urban Kenyan hospital"); diabetes and depression analysed together with HIV.
Mendenhall & Norris [34] (C)	2015	Diabetes, Depression, HIV, NCDs	Direct	Direct	Diabetes and depression analysed together with HIV in women with diabetes, within the syndemic suffering framework. Shared dataset C.
Page-Reeves et al. [40]	2019	Diabetes, Depression	Direct	Direct	Framework named in the title ("Addressing Syndemic Health Disparities Among Latin Immigrants Using Peer Support"); diabetes and depression are the two conditions addressed.
Saxena & Mendenhall [42] †	2022	Hypertension, Type 2 diabetes, Functional disability; Depression (discussed, not analysed)	Indirect	Direct	Criterion 3: syndemic theory is the stated framework (title). Criterion 2: the analysis concerns diabetes, hypertension and disability; depression was not analysed ("We did not use the self-report for depression because there were too few responses", p. 8). Its coexistence with diabetes is part of the syndemic framing and of the discussion: "the lowest-income communities face compounded social stress, untreated depression, and poor healthcare access alongside these clustered 'metabolic' conditions" (abstract, p. 1); "These clustered conditions often co-occur with obesity, depression, anxiety, and heightened social, emotional, and family stress" (p. 2); "low-income people with diabetes faced more compounded social stress, financial insecurity, depression, and delayed care-seeking" (p. 8).
Weaver & Mendenhall [32] (B)	2014	Diabetes, Depression	Direct	Direct	Title names both conditions and the framework: "Applying Syndemics and Chronicity: Interpretations from Studies of Poverty, Depression, and Diabetes". Shared dataset B.

Study [reference]	Year	Conditions examined	Criterion 2: diabetes–depression coexistence	Criterion 3: syndemic framework	Basis for the decision (supporting passage and page where eligibility was questioned)
Zuñiga et al. [53] †	2022	HIV, Diabetes, Treatment adherence, Depression, Quality of life, Self-management	Direct	Indirect	Criterion 2: depression is a measured variable (PHQ-9) in people living with HIV and type 2 diabetes: "An increase in lagged depression was associated with a small decrease in HRQoL" (abstract, p. 1); "people living with HIV or diabetes report levels of depression higher than the general population's" (p. 3). Criterion 3: syndemic concepts frame the population and the interpretation, while the analysis tests a self-management model: "Our target population comprised people who experience the dual diagnosis of HIV and T2DM, which can be described as currently presenting a syndemic" (p. 3); "The syndemic model suggests that variables not traditionally addressed in self-management interventions will need to be investigated, including stigma, racism, and violence" (p. 8). "Syndemic" is a keyword (p. 2).
Study protocols (n = 2)					
Awoke et al. [54]	2025	Diabetes, Depression, Functional disability	Direct	Direct	Title names both conditions and the framework: "Syndemics of depression, sick role and activation status among newly diagnosed adults with diabetes mellitus…". Study protocol (no results yet).
Friedman et al. [55] †	2024	HIV/AIDS, NCDs (diabetes, hypertension, dyslipidaemia), Depression	Indirect	Direct	Study protocol (no results yet). Criterion 3: syndemic theory is the stated framework (title; abstract, p. 1). Criterion 2: depression is a component of the "psychosocial syndemic (stress, depression, violence victimisation and substance use)" (abstract, p. 1) and is measured with the CES-D-10 (p. 4); diabetes is a component of the "NCD syndemic prevalence (diabetes, dyslipidaemia and hypertension)" (p. 6). The protocol hypothesises that "intersectional stigma and the psychosocial syndemic act as serial mediators, contributing to NCD syndemic incidence and prevalence disparities by social position" (p. 2). Both conditions are in the same model but are not analysed as a dyad.
Editorials / short articles (n = 4)					
Arena et al. [56]	2022	COVID-19, Depression, Overweight/Obesity, Diabetes	Direct	Direct	Editorial (conceptual). Framework named in the title ("…reside in the same Syndemic City"); depression and diabetes discussed together.
Gallo et al. [57]	2021	Depression, Diabetes, Cognitive changes	Direct	Direct	Editorial (conceptual). Framework named in the title ("…Preventing the Syndemic of Depression and Medical Comorbidity"); diabetes is among the comorbidities discussed.
Mendenhall et al. [58]	2022	COVID-19, Diabetes, Depression, Musculoskeletal condition	Direct	Direct	Perspective (conceptual). Framework named in the title ("Syndemics and clinical science"). One of its two case examples is the diabetes–depression syndemic: "We also explore two case examples: the first involving depression and diabetes in Mexican immigrants in the United States" (p. 1359); "the close biological and psychological links between depression and type 2 diabetes" (p. 1360); "their diabetes was entangled in a feedback loop with traumatic memories, family stress, chronic financial uncertainty and untreated depression" (p. 1361).

Study [reference]	Year	Conditions examined	Criterion 2: diabetes–depression coexistence	Criterion 3: syndemic framework	Basis for the decision (supporting passage and page where eligibility was questioned)
Ricceri [59]	2024	Multimorbidity among migrant populations; Diabetes, Depression (VIDDA syndemic)	Direct	Direct	Short article (conceptual), in Italian with an English abstract. Framework named in the title ("…dal punto di vista sindemico"). The diabetes–depression syndemic is its central example: "the most interesting in this area is the one concerning the increased frailty due to the interaction among diabetes, depression, immigration, and social distress, called VIDDA" (English abstract, p. 21); the VIDDA studies show how these variables affect "il rischio di depressione e di diabete" (p. 23).
Books (n = 2)					
Mendenhall [37]	2019	Syndemic suffering, Diabetes, Depression	Direct	Direct	Book (ethnography): "Rethinking Diabetes: Entanglements with trauma, poverty, and HIV"; diabetes and depression analysed together within syndemic theory.
Mendenhall [12] (B)	2012	Diabetes, Depression	Direct	Direct	Book. Title names both conditions and the framework: "Syndemic suffering: social distress, depression, and diabetes among Mexican immigrant women". Shared dataset B.
Theses (n = 1)					
Fox [60]	2016	Diabetes, Depression, Syndemic suffering	Direct	Direct	Thesis. Title names both conditions and the framework: "Diabetes, Depression and Syndemic Suffering among African American Patients…".

Reference numbers in square brackets are those of the reference list of the article; publications are listed in the same order as in Table 1. † Met one core eligibility criterion indirectly. Shared study or dataset: A, Brazil National Health Survey 2013 [30,31]; B, Chicago / original VIDDA data [12,13,32]; C, Birth to Twenty (Bt20) cohort, Johannesburg [13,33–35]; publications sharing a letter are not independent evidence. Page numbers are those printed in the version of the publication consulted; for Lerman Ginzburg [52] (online-first version, unnumbered) and Zuñiga et al. [53] (author manuscript in PubMed Central) they are the pages of the PDF. CHD, coronary heart disease; NCDs, non-communicable diseases; TB, tuberculosis; PTSD, post-traumatic stress disorder; T2DM, type 2 diabetes mellitus; HRQoL, health-related quality of life; PHQ-9, Patient Health Questionnaire; CES-D-10, Center for Epidemiologic Studies Depression scale; VIDDA, violence, immigration, depression, diabetes and abuse. Source: authors' own elaboration.

Part B. Reports excluded at full-text assessment (n = 13)

Each report has one primary reason for exclusion, which corresponds to the categories shown in the PRISMA flow diagram (Figure 2): no coexistence or interaction between diabetes and depression (n = 12) and not relevant to the syndemic concept (n = 1).

#	Authors (year)	DOI	Conditions studied	Criterion 2: diabetes–depression coexistence	Criterion 3: syndemic framework	Primary reason for exclusion (category in Figure 2)
1	Kim et al. (2026)	10.1136/bmjopen-2025-109258	Social isolation, Mental health, Diabetes, Health behaviours (tobacco use, alcohol consumption, physical activity)	Not met: depression is not a distinct condition (mental health measured generically)	Yes	No coexistence/interaction between diabetes and depression
2	Sperling et al. (2026)	10.5334/gh.1541	Cardiovascular disease, Multiple long-term conditions (including diabetes, chronic kidney disease, obesity and mental health disorders)	Not met: diabetes and depression are not analysed as distinct conditions	No	No coexistence/interaction between diabetes and depression

#	Authors (year)	DOI	Conditions studied	Criterion 2: diabetes–depression coexistence	Criterion 3: syndemic framework	Primary reason for exclusion (category in Figure 2)
3	Zainab & Kodali (2026)	10.1007/s44192-026-00419-2	Depressive symptoms, Mental healthcare use	Not met: diabetes is not studied	No	Not relevant to the syndemic concept
4	Abdul-Mutakabbir & Abdul-Mutakabbir (2025)	10.1007/s40121-025-01188-1	Non-communicable diseases, Social deprivation, Multidrug-resistant infections	Not met: neither condition is analysed as a distinct condition	Not explicit	No coexistence/interaction between diabetes and depression
5	Bal et al. (2025)	10.1371/journal.pone.0319073	Non-communicable diseases, HIV	Not met: depression is not studied	Yes	No coexistence/interaction between diabetes and depression
6	Mino-León et al. (2025)	10.1016/j.arcmed.2023.102869	Chronic diseases; socioeconomic, health, behavioural and functional indicators	Not met: diabetes and depression are not analysed as distinct conditions	Yes	No coexistence/interaction between diabetes and depression
7	Oresnik et al. (2025)	10.1016/j.socscimed.2025.117994	Gestational diabetes mellitus, Anxiety and mood disorders, Food insecurity	Not met: gestational diabetes; depression is not a distinct condition	Yes	No coexistence/interaction between diabetes and depression
8	Salisu et al. (2025)	10.3389/fpsyt.2025.1711836	Serious mental illness, Cardiovascular disease	Not met: diabetes is not studied	Not explicit	No coexistence/interaction between diabetes and depression
9	Chichetto et al. (2022)	10.1007/s10461-021-03327-4	Unhealthy alcohol use, Smoking, Depressive symptoms, Cardiovascular disease	Not met: diabetes is not studied	Yes	No coexistence/interaction between diabetes and depression
10	Slagboom et al. (2022)	10.7189/jogh.11.04029	Psychological distress, Cardiometabolic conditions, Musculoskeletal pain	Not met: neither condition is analysed as a distinct condition	Not explicit	No coexistence/interaction between diabetes and depression
11	Weaver & Kaiser (2022)	10.1016/j.socscimed.2020.113304	Poverty, Mental health, Food insecurity, Type 2 diabetes	Not met: depression is not a distinct condition (mental health measured generically)	Not explicit	No coexistence/interaction between diabetes and depression
12	Chudasama et al. (2021)	10.1186/s12879-021-06600-y	Multimorbidity	Not met: neither condition is analysed as a distinct condition	Not explicit	No coexistence/interaction between diabetes and depression
13	Jones et al. (2021)	10.1161/JAHA.120.019340	Education, Obesity, Cigarette smoking, Depressive symptoms	Not met: diabetes is not studied	Not explicit	No coexistence/interaction between diabetes and depression

Criterion 3: “Yes”, the syndemic concept is the stated framework; “No”, it is not used; “Not explicit”, the publication does not state a syndemic framework. Source: authors’ own elaboration.
